# Non-linear finite element modeling of damages in bridge piers subjected to lateral monotonic loading

**DOI:** 10.1038/s41598-023-39577-6

**Published:** 2023-08-18

**Authors:** Aizaz Ahmad, Awais Ahmed, Mudassir Iqbal, Syed Muhammad Ali, Ghufranullah Khan, Syed M. Eldin, Ahmed. M. Yosri

**Affiliations:** 1 Department of Civil Engineering, University of Engineering & Technology, Peshawar, 25120 Pakistan; 2 Oslo Metropolitan University, 0166 Oslo, Norway; 3 State Key Laboratory of Ocean Engineering, Shanghai Key Laboratory for Digital Maintenance of Buildings and Infrastructure, School of Naval Architecture, Ocean & Civil Engineering, Shanghai Jiao Tong University, Shanghai, 200240 China; 4 School of Civil Engineering, Tianjin University, Tianjin, China; 5 Center of Research, Faculty of Engineering, Future University in Egypt, New Cairo, 11835 Egypt; 6 Department of Civil Engineering, College of Engineering, Jouf University, Sakakah, Saudi Arabia; 7 Civil Engineering Department, Faculty of Engineering, Delta University for Science and Technology, Belkas, Egypt

**Keywords:** Engineering, Civil engineering

## Abstract

Bridges are among the most vulnerable structures to earthquake damage. Most bridges are seismically inadequate due to outdated bridge design codes and poor construction methods in developing countries. Although expensive, experimental studies are useful in evaluating bridge piers. As an alternative, numerical tools are used to evaluate bridge piers, and many numerical techniques can be applied in this context. This study employs Abaqus/Explicit, a finite element program, to model bridge piers nonlinearly and validate the proposed computational method using experimental data. In the finite element program, a single bridge pier having a circular geometry that is being subjected to a monotonic lateral load is simulated. In order to depict damages, Concrete Damage Plasticity (CDP), a damage model based on plasticity, is adopted. Concrete crushing and tensile cracking are the primary failure mechanisms as per CDP. The CDP parameters are determined by employing modified Kent and Park model for concrete compressive behavior and an exponential relation for tension stiffening. The performance of the bridge pier is investigated using an existing evaluation criterion. The influence of the stress–strain relation, the compressive strength of concrete, and geometric configuration are taken into consideration during the parametric analysis. It has been observed that the stress–strain relation, concrete strength, and configuration all have a significant impact on the column response.

## Introduction

A bridge is an integral component of the transportation network. Unlike other highway structures, its failure for any reason can have serious economic and life consequences. Among the different natural disasters, earthquakes pose more danger to bridges due to their unpredictable nature. More than 40 deaths and $1.8 billion loss occurred in the Loma Prieta earthquake due to bridge damages^1^. The Chi-Chi earthquake in 1999, which had a magnitude of 7.3, damaged or caused the collapse of about 200 bridges^2^. Similarly, in the 2008 Wenchuan earthquake, 6140 bridges either collapsed or suffered damages, and the transportation system suffered a loss of 67 billion RMB^3^. Significant changes were made in the seismic design of bridges in the Chinese code after the 2008 Wenchuan earthquake. In the 2005 Kashmir earthquake, many bridges suffered damages, with some experiencing damages beyond repair. A survey of the existing bridge stock of Pakistan was conducted in the wake of the 2005 earthquake^4^. It was ascertained that, out of the 6000 bridges surveyed, almost 67% of bridges on the major highways were constructed in the mid-80s. In addition, the official bridge design code of Pakistan, West Pakistan Code of Highways Practice, is adopted from the 1965 version of AASHTO, which was developed before the recent advances in the seismic design of bridges. All these factors collectively contributed to the poor performance of the bridges in the 2005 earthquake. Furthermore, it is also evident that given the aforementioned factors, the existing bridge stock of Pakistan is seismically vulnerable.

An experimental test for the lateral response of the structure can be carried out with four distinct methods^5^. However, two test techniques, dynamic shake table testing and quasi-static reverse cyclic testing, are often used to evaluate the dynamic behavior of bridge piers. Experimental studies are significant in the seismic assessment of bridge piers, but the experimental tests cannot always be performed with the full-scale models due to the limitation imposed by space, time and equipment requirements^6^. Ali^7^ and Iqbal^8^ performed quasi-static cyclic testing on scaled-down models to study the behavior of low-strength concrete bridge piers. Deng et al.^9^ and Prakash et al.^10^ studied the effect of combined axial share, bending, and torsion on the bridge piers. Schoettler et al.^11^ and Sakai and Unjoh^12^ analyzed the effect of dynamic excitation on the behavior of bridge piers. Hose and Seible^13^ developed a damage assessment and performance evaluation criteria for post-earthquake evaluation of bridge piers. A 5-level qualitative performance evaluation criteria was presented and verified by a series of experimental tests on multiple bridge piers with different geometries.

For complex engineering problems variety of numerical and machine learning models are employed^14,15^. Generally, nonlinear behavior of bridge piers can be assessed using numerical simulations. The three main numerical techniques employed in this regard are lumped plasticity, distributed plasticity (fiber-section approach) and finite element-based model approach (FEM). The FEM-based approach has been found to predict the non-linear behavior of concrete structures more realistically. In addition to this FEM based approach also can predict a detailed behavior incorporating a more exact failure mechanism of structure^16^. Deng et al.^9^ used Abaqus to analyze rebar stains under the influence of axial, bending shear, and torsion. Modeling concrete was achieved using the integrated concrete damaged plasticity model (CDP). Kishi et al.^17^ investigated the effect of the softening branch of concrete compressive behaviour on bridge pier damages. Using Abaqus CDP, Dawood et al.^18^ investigated the behaviour of segmentally post-tensioned concrete bridge piers. The performance of Abaqus CDP under reverse cyclic analysis of bridge piers was investigated by^19^. CDP with viscous regularizing was compared to the SDP (softened damaged model given by^20^ and it was determined that SDP performs better than CDP in reverse cyclic analysis while employing an implicit solver in reverse cyclic loading at high strain rates.

Given the limitations imposed by the experimental testing of bridge piers due to the non-availability of resources, Finite element method plays an important role in the non-linear analysis and assessment of bridge piers. Concrete Damaged Plasticity, CDP, which is very frequently utilized for concrete modeling has many important parameters that can significantly influence the outcome of the study. These parameters are not investigated thoroughly in the available research. Furthermore, numerical studies are based on certain numerical equations and solution techniques that provide approximate solutions. Hence, the selection of analysis procedure can be influential on the results which is mostly overlooked. In this regard this study focuses on the non-linear modeling of bridge piers under lateral loading. With the help of existing experimental data, this work attempted to numerically validate the lateral response of a bridge pier and check the effect of various parameters such as dilation angle, stress strain relation, pier geometry and configuration on the bridge pier response. Practically, such a computational scheme can be used for case study research, design, or retrofit purpose without the need for rigorous experimental work.

A single circular bridge pier based on the study conducted by^7^ was selected. The pier is ¼ scaled down model of an existing bridge piers, having 12-in. diameter and 146-in. height. The concrete used in the pier is 2400 psi with 26 rebars having yield strength of 53 ksi. The pier is modeled using the finite element software, Abaqus/explicit. The pier is modeled with an initial mesh size of 2 in. and loading step of 60 s and a CDP dilation angle of 40. A monotonic displacement-type loading is applied on the pier and the response is compared with the experimental backbone curve. The validation of the model is carried out by comparing the backbone curve of the real experimental data of^7^. Following validation, more investigations are carried out to determine how various parameters affect the behavior of single-column bridge piers. These factors include the concrete stress–strain relation, concrete strength, and dilation angle. In addition, the numerical scheme presented here is utilized to model multiple pier geometries and a twin-column configuration.

To model the non-linear behavior of concrete, CDP in Abaqus is utilized. Modified Kent and Park model and fracture energy-based criterion are employed to model the compressive and tensile behavior of concrete. Rebars are modeled using the bi-linear elastoplastic relation. Abaqus explicit solver is employed for the nonlinear modeling and the effect of step-time mesh size and configuration is investigated. The manuscript is outlined such that the following section presents a detailed description of the experimental test followed by the numerical modeling process using the Abaqus. A brief overview of the Abaqus CDP is given in the subsequent section. The effect of step time, mesh size and configuration is discussed afterward and the effect of the different parameters is also discussed. Finally, the conclusions based on the study conducted in this paper are presented.

## Methodology

### Experimental model

After the 2005 earthquake, an extensive survey was carried out in the affected areas of Pakistan^7^, where many bridges suffered damage. A total of 90 bridges were inspected, out of which 14 were significantly affected by earthquake. In addition to the visual inception of the bridge, core samples were also taken from the bridge substructures for material quality assessment. Strength as low as 13.8 MPa (2000 psi) was observed during the on-field testing, with average strength up to 17.2 MPa (2494 psi). In light of the above, an experimental test was carried out to assess the seismic behavior of low-strength concrete bridge piers.

**Figure 1:**
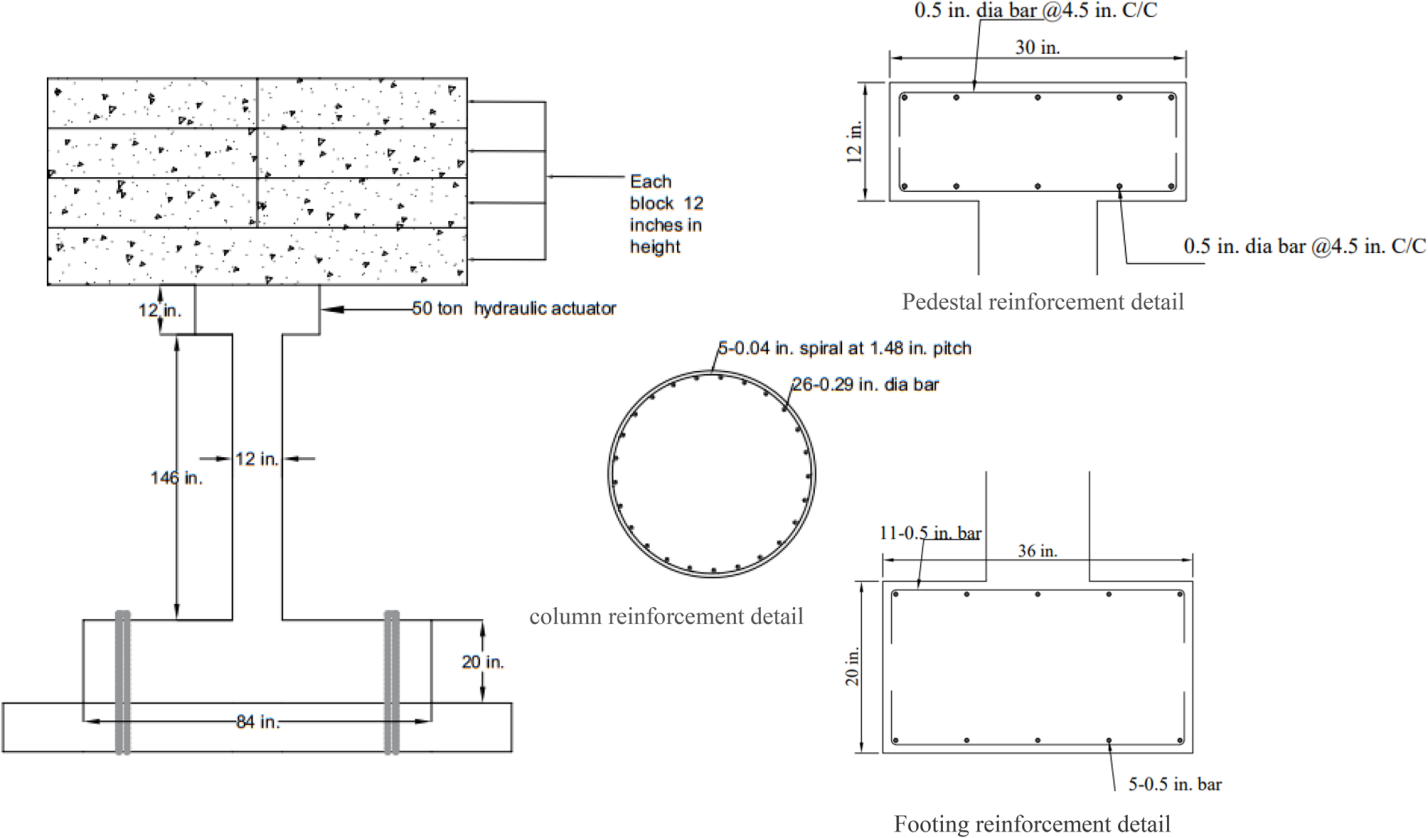
Geometry of experimental bridge pier.

**Figure 2:**
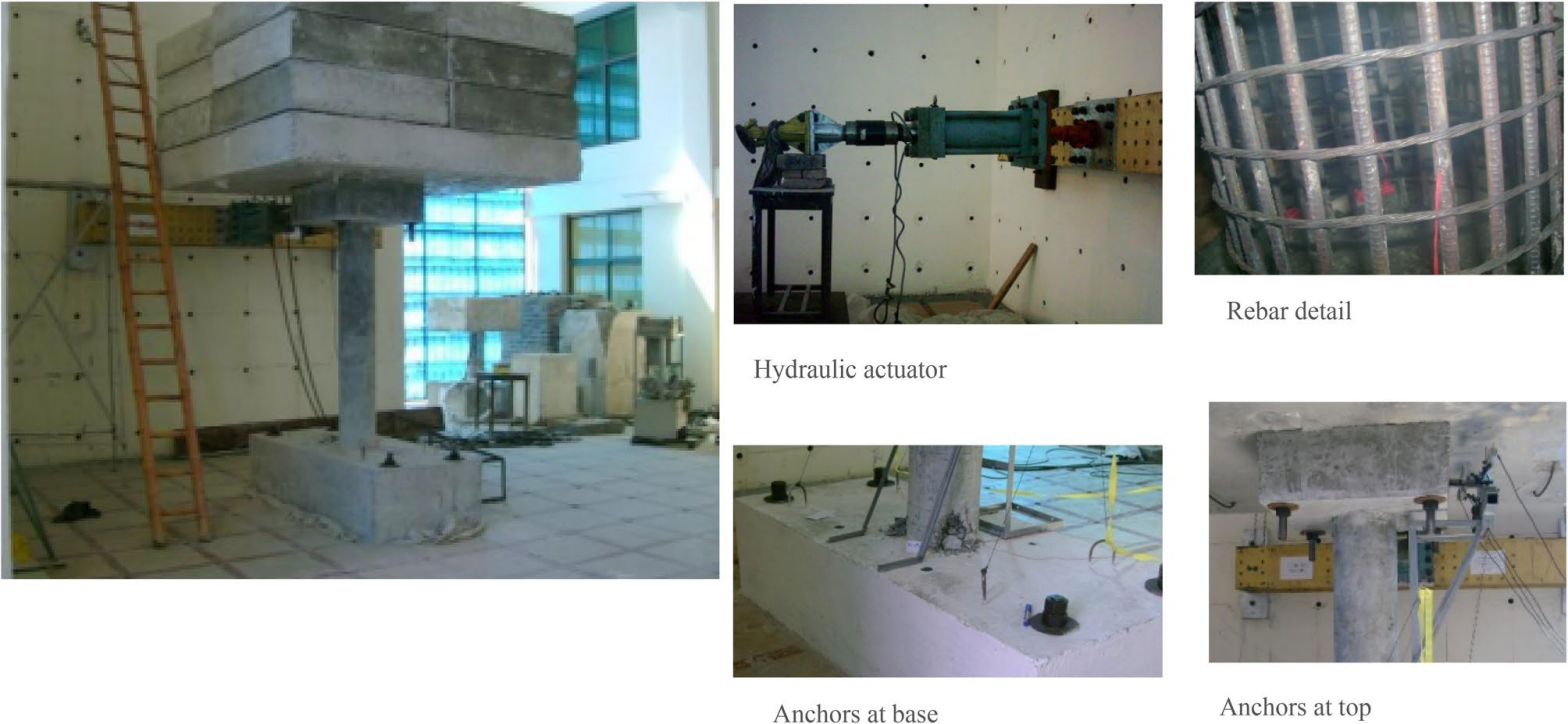
Bridge pier experimental setup.

The geometry of the bridge pier is shown in Fig. 1 and the experimental setup is shown in Fig. 2. Due to the limitation of equipment and testing protocol, scaled-down column was built with a scale factor ¼ for the column. The column had a clear height of 146 in. At the top of the column, a reinforced concrete pedestal was provided for the placement of the dead load. The column was erected from a base (Length = 84 in, Width = 36 mm, Depth = 20 in). The base was connected to the strong floor through four high-strength bolts. Typical superstructure weight was determined to be 310 tons on a single bridge pier. In the experimental test, concrete blocks were used as superstructure load to account for a scaled-down values of 19.38 tons.

The cross-sectional details are shown in Fig. 1, which contains 0.29 in. rebars. Due to the unavailability of smaller diameters, 5 number of 0.04 in.. rebars wounded around each other were used as confining rebars. The main rebars were confined only within the clear height of the column with a pitch of 1.48 in. The footing was reinforced with 11 number of 0.5 in. rebars provided both at the top and bottom in the transverse direction Lap is not provided in footing bars due to the availability of 40 feet (480 in.) bars. 5–0.5 in. rebars were also provided in the longitudinal direction along with the transverse bars.

Table 1 shows the properties of the material used in the experimental test^7^. The 4 × 4 feet slabs for the dead load were reinforced with 3 number of 0.5 in. diameter bars in both directions. The single 8 × 8 foot slab was reinforced with 0.5 in. rebars having a spacing of 5 in. The top pedestal was reinforced with 0.5 in. grade 60 rebars at 4.5 in. center to center spacing.

**Table 1:** Properties of material of experimental column.

Material property	Value
Concrete compressive strength	2400 psi
Modulus of rupture	674 psi
Elastic modulus	2798 ksi
Yield strength of rebar	53 ksi
Ultimate strength of rebar	70.4 ksi
Yield strain of rebar	0.0018 in/in
Ultimate strain of rebar	0.019 in/in

The equipment’s used for the testing included a hydraulic actuator having 50-ton capacity. Another hydraulic actuator having a 25-ton capacity was also utilized to perfrom the test in a cyclic quasi static manner. Both of the actuator had a speed of 0.256in./sec. Load cells were attached to the actuator to measure the applied forces. The hydraulic jacks were mounted on structural framing having adjustable girders. In addition to this the displacement at the load application point was measured with the help of linear variable deferential transformer (LVDT) and string potentiometer. A data acquisition system (UCAM-70) was used for the quasi-static testing data having 30 channels. The anchoring of the column base was achieved through four, 44-in.-long anchor having diameter of 1.75 in. Similarly, to anchor the slabs with the top of the column the four, 27-in.-long anchor having diameter of 1 in. were used.

The column was subjected to quasi-static reverse cyclic loading. The loading protocol adopted for the column consisted of three cycles per drift level. A total of 37 cycles were applied, with a maximum of 4% drift in the final cycle. These loads were applied through a 50-ton Hydraulic actuator. The total time taken by the test was 3600 s.

### Finite element modeling

Abaqus was used for the bridge pier's numerical modeling. Abaqus is a general-purpose finite element method (FEM) program that can evaluate static and dynamic problems in concrete structures^22^. It offers both implicit and explicit analysis options for non-linear modeling. Modeling in the Abaqus is usually carried out using the sequence: geometry, material, assembly, contacts, loads and boundary condition, analysis, and post-processing. Some of the limitations of Abaqus modeling include that it needs high computational power for three dimensional simulations. Rebar rupture and concrete cracking cannot be explicitly modeled as well. A higher mesh size could overestimate the damage region while using CDP. Additionally, the CDP concrete model does not shows any convincing results in reverse cyclic condition^19^. Some of the potential sources of errors are inaccurate material data input for the CDP model, inaccurate constraints between the different concrete components and highly distorted mesh elements. Abaqus offers two main material constitutive models for concrete Structures: concrete damaged plasticity (CDP), which is available both in Abaqus/Standard and Abaqus/explicit and smeared cracking model used only in Abaqus/Standard. CDP was employed for the nonlinear modeling of concrete. The concrete elements were meshed using the C3D8 elements, which has 2 × 2 × 2 integration scheme. Rebars were model using the two-node linear truss T3D2 element due to their result accuracy and minimum computational effort^21^. The embedded constraint was employed for the interaction between rebars and concrete, while the interaction between the concrete surfaces was modeled through tie constraints. To avoid the relative movement of the base, the Encastre boundary condition was applied on the base of the footing. Rebars material was modeled using the simple bilinear elastoplastic law.

**Figure 3:**
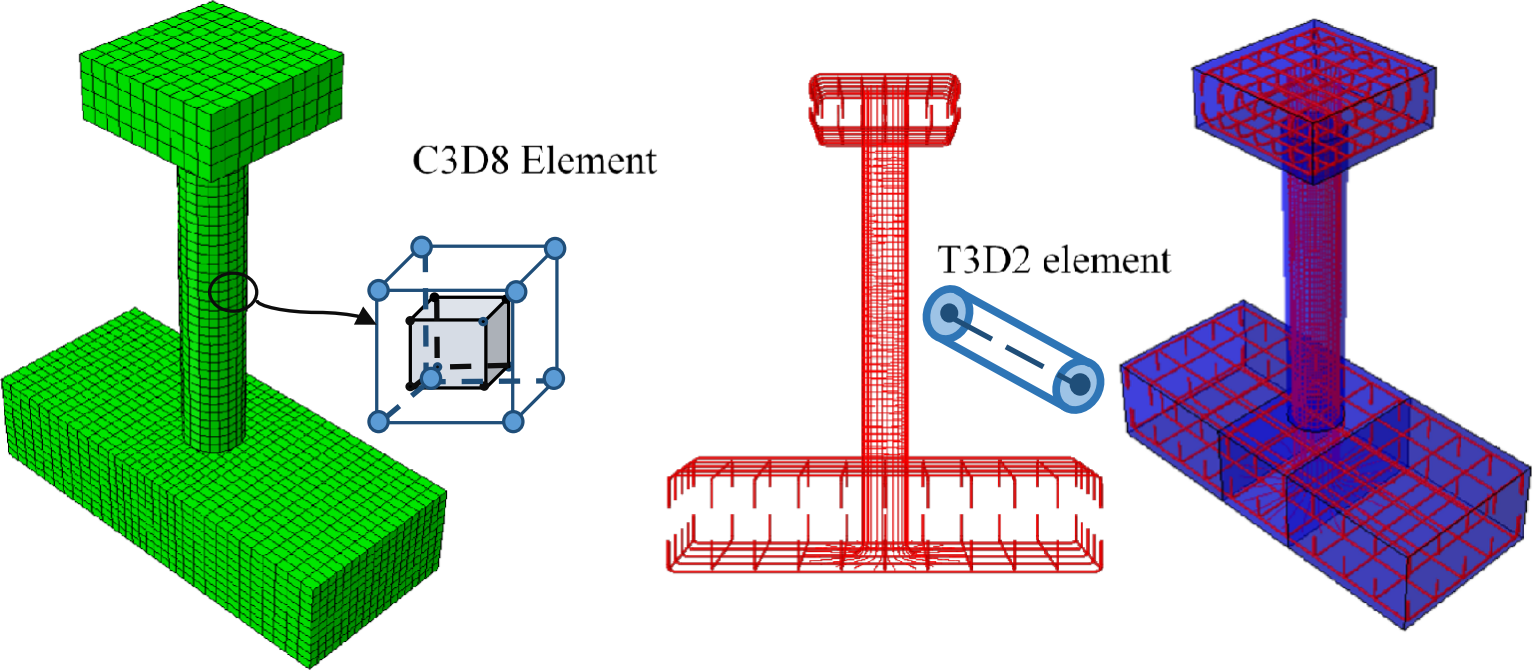
Concrete and Rebar components of the model with assembly.

Material degradation and failure leads to severe convergence difficulties in implicit analysis which involves inversion of stiffens matrix. An example of material degradation is concrete cracking which leads to the formation of negative stiffness matrix^22^. Additionally, implicit analysis was employed initially, however, the computational time could not be determined because of the convergence errors which lead to simulation stoppage. Generally, with regard to the computational efficiency, the analysis time in Abaqus/explicit is proportional to the number of elements which in our case is 9094 elements. While, the analysis time in implicit solver is roughly proportional to the square of number of degrees of freedom (38,095 d.o.f in the model). Hence, for the analysis, Abaqus explicit solver was employed with double precision. The model developed in Abaqus is shown in Fig. 3.

### Overview of concrete damage plasticity (CDP)

**Figure 4:**
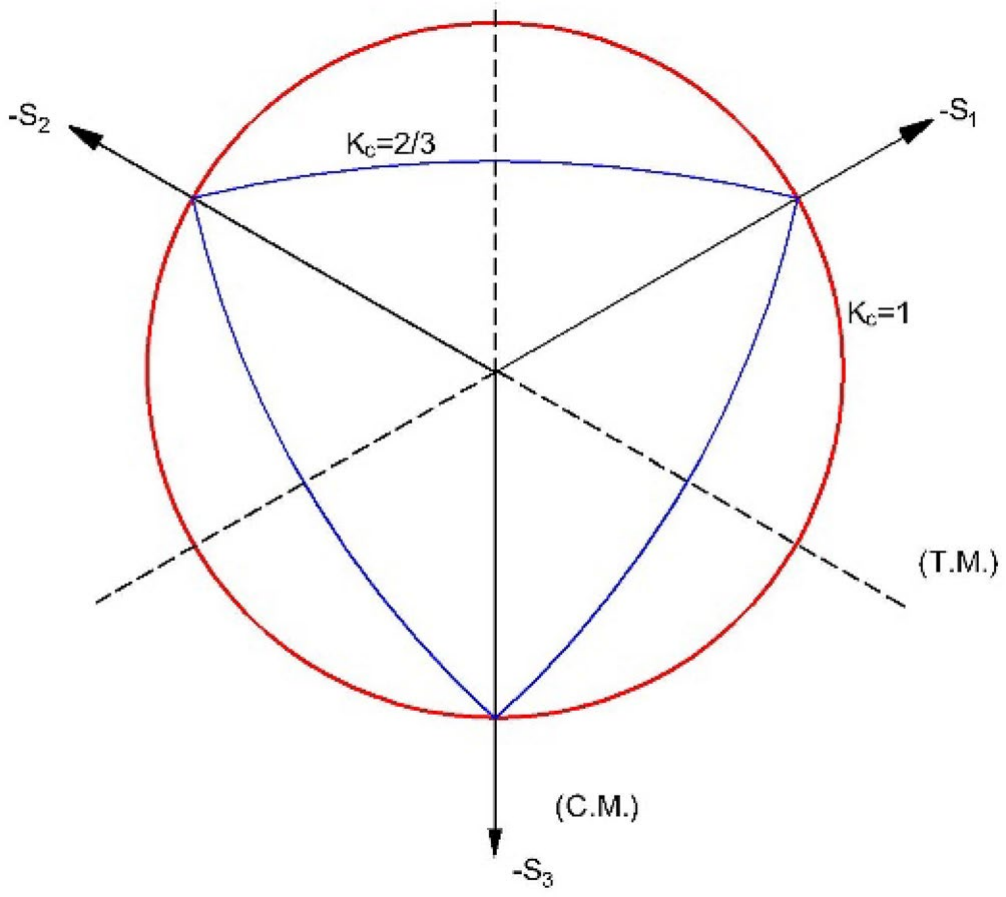
Yield function in deviatoric plane.

To model damages observed during the experiment, Abaqus CDP was employed for modeling concrete. The damage plasticity model for concrete, CDP, first given by Lubliner et al.^23^ and further modified by Lee and Fenves^24^, is based on the model of Continuum Plasticity damaged. The model considers cracking in tension and crushing in compression as the main failure mechanism of concrete. Similar to all plasticity based models, CDP requires a yield function, flow rule and hardening rule. The yield function in the deviatoric plane is shown in Fig. 4. The yield function adopted by CDP is given as Eq. (1).

1 $$F=\frac{1}{1-a}(\overline{q }-3\alpha \overline{p }+\beta \left({\varepsilon }^{\sim pl}\right)\langle {\widehat{\tilde{\sigma }}}_{max}\rangle -\gamma \langle {\widehat{\tilde{\sigma }}}_{max}\rangle )-{\sigma }_{c}\left({\varepsilon }^{\sim pl}\right)=0$$

The effective part of hydrostatic stress $\stackrel{\mathrm{-}}{\text{p}}$ and the effective Mises equivalent stress $\overline{q }$ are provided as Eqs. (2) and (3), respectively.

2 $$\overline{p }=-\frac{1}{3}\tilde{\sigma }: {\rm I} $$

3 $$\overline{q }=\sqrt{\frac{3}{2}\overline{S }:\overline{S} }$$

where $\tilde{\sigma }={D}_{0}^{el}:\left(\varepsilon -{\varepsilon }^{pl}\right) (\mathrm{effective stress})$, $\overline{S }=\overline{p}\boldsymbol{\rm I }+\tilde{\sigma }$(deviatoric part of the effective stress tensor), ${\widehat{\tilde{\sigma }}}_{max}$ (algebraically maximum eigenvalue of $\tilde{\sigma }$).

The other parameters of the function are Eqs. (4)–(6)

4 $$a=\frac{\left(\frac{{\sigma }_{b0}}{{\sigma }_{c0}}\right)-1}{2\left(\frac{{\sigma }_{b0}}{{\sigma }_{c0}}\right)-1};0\le \alpha \le 0.5$$

5 $$\beta =\frac{{\tilde{\sigma }}_{c}\left({\widetilde{\varepsilon }}_{c}^{pl}\right)}{{\tilde{\sigma }}_{t}\left({\widetilde{\varepsilon }}_{t}^{pl}\right)}\left(1-a\right)-(1+a)$$

6 $$\gamma =\frac{3.(1-{K}_{C})}{2.{K}_{C}-1}$$

Typical experimental values of the ratio $\frac{{\sigma }_{b0}}{{\sigma }_{c0}}$ (Ratio of biaxial compressive strength of concrete to uniaxial strength) for concrete are in the range from 1.10 to 1.16^23^. ${\tilde{\sigma }}_{c}$ and ${\tilde{\sigma }}_{t}$ are the effective tensile and compressive cohesion stresses, respectively. The second stress invariant on the tensile meridian to the compressive meridian is measured as ${(K}_{C}$). Tensile meridian (TM) is the line that passes through the point corresponding the to the stress states: ${\widehat{\tilde{\sigma }}}_{max}$= ${\widehat{\tilde{\sigma }}}_{1}$> ${\widehat{\tilde{\sigma }}}_{2}$= ${\widehat{\tilde{\sigma }}}_{3}$ while Compressive meridian (CM) is the line joining the point that corresponds to the stress states such that ${\widehat{\tilde{\sigma }}}_{max}$= ${\widehat{\tilde{\sigma }}}_{1}$= ${\widehat{\tilde{\sigma }}}_{2}$>${\widehat{\tilde{\sigma }}}_{3}.$

CDP's plastic potential function is a non-associated Drucker-Prager hyperbolic function defined by Eq. (7).

7 $$G=\sqrt{ (\epsilon .{\sigma }_{t0}.\mathrm{tan\psi })+{\overline{q} }^{2} }-\overline{p }.\mathrm{tan}\psi $$

**Figure 5:**
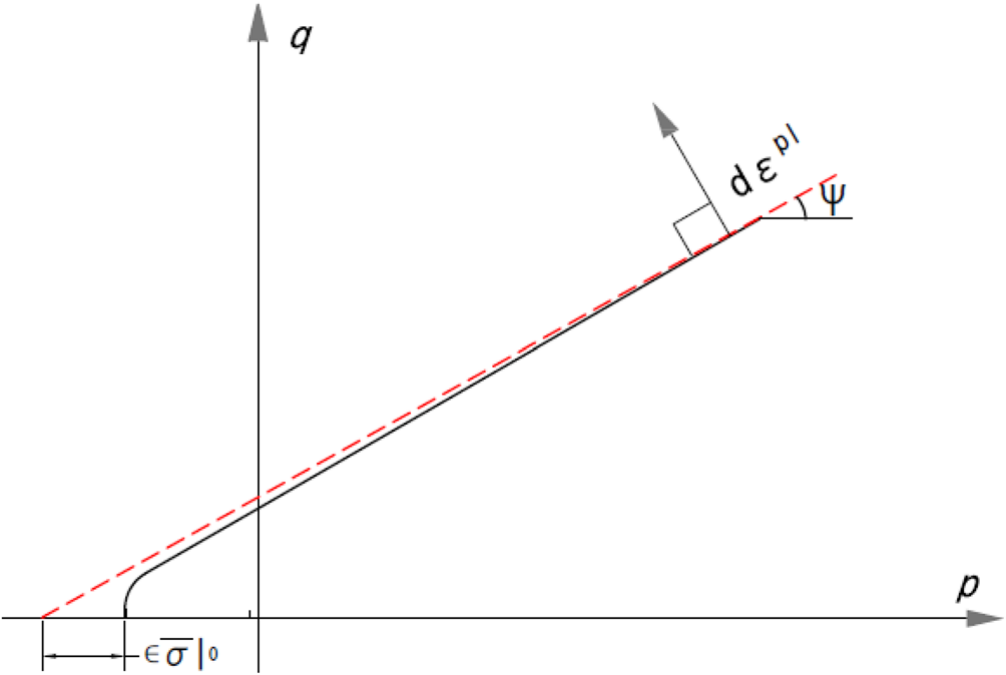
Dilation angle and eccentricity in meridional plane.

The eccentricity ϵ and dilation angle ($\psi$) are depicted in Fig. 5. ϵ—eccentricity—is the eccentricity that gives the rate at which the plastic potential function approaches the asymptote. $\psi$—dilation angle—refers to the change in volume of the material, mainly, due to the deviatoric part of the plastic strain tensor. Hence it can also be defined as the ratio of the deviatoric part of the plastic strain tensor to the volumetric part of the plastic stain tensor A value of 40 for the dilation angle was initially chosen based on the reported study^25^. Values for the rest of the parameters are adopted from the Abaqus manual, which are commonly employed and also recommended by^25^. Table 2 lists the values of the various CDP parameters that were used in the initial modeling. In order to prevent convergence issues, the CDP also includes viscoplastic regularization for implicit analysis. This is accomplished by including the viscous parameter (μ). The parameter Viscosity (μ) is taken as zero due to the application of an explicit solver. The effect of the ϵ, $\psi$ and ${(K}_{C}$) on the yield function can be found in detail in^26^.

**Table 2:** CDP parameters used for the numerical modeling.

CDP parameters	$\psi (^\circ )$	$\epsilon$	($\frac{{\sigma }_{b}}{{\sigma }_{c}}$)	${K}_{C}$	*Μ*
Value	40	0.1	1.16	0.667	0

The concrete-damaged plasticity requires plastic strains and stress as input in compression and tension to model concrete behavior. The inelastic strain and stress from the stress–strain relation of concrete are then converted to plastic strains and stress by CDP. The inelastic strains can be either calculated from the experimental stress–strain curve or an analytical stress–strain relation can be used to get these values. The other parameters that are required by the CDP to model concrete are the Poisson ratio and modulus of elasticity. The modulus of elasticity was calculated as per (ACI 318-02, 2001) and the value of 2796 ksi (19,277 MPa) was used for the numerical modeling and a value of 0.2 was used for the Poisson ratio.

**Figure 6:**
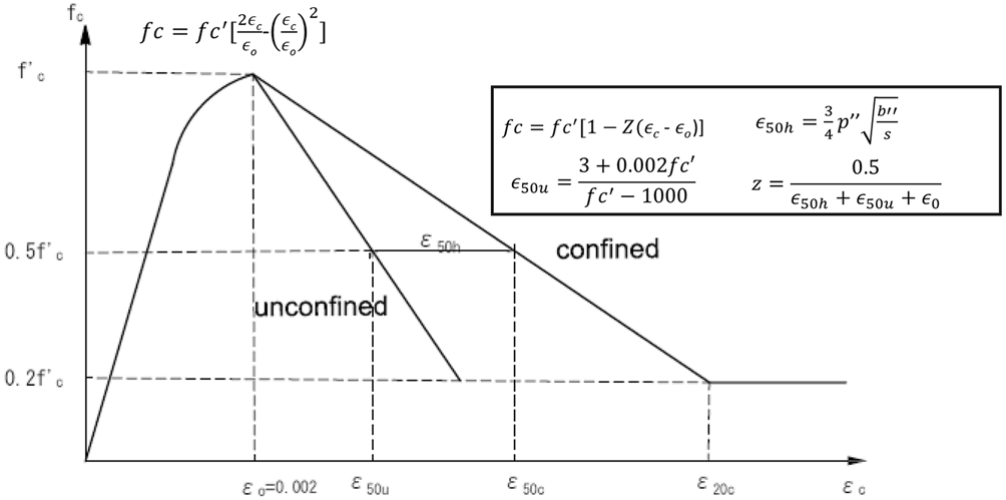
Kent and Park model for concrete compressive behavior.

In compression, the concrete behaves elastically up to a certain limit, mostly in the range of 40–50%. After the initial elastic response, the inelastic region is characterized by an initial stress hardening region up to the peak stress, followed by strain softening in the post-peak branch. The modified Kent and Park^27^ model shown in Fig. 6 is utilized to model the uniaxial stress–strain behavior of concrete. The model consists of two constitutive relations for concrete: confined and unconfined. The confined relation is primarily employed for the softening branch.

**Figure 7:**
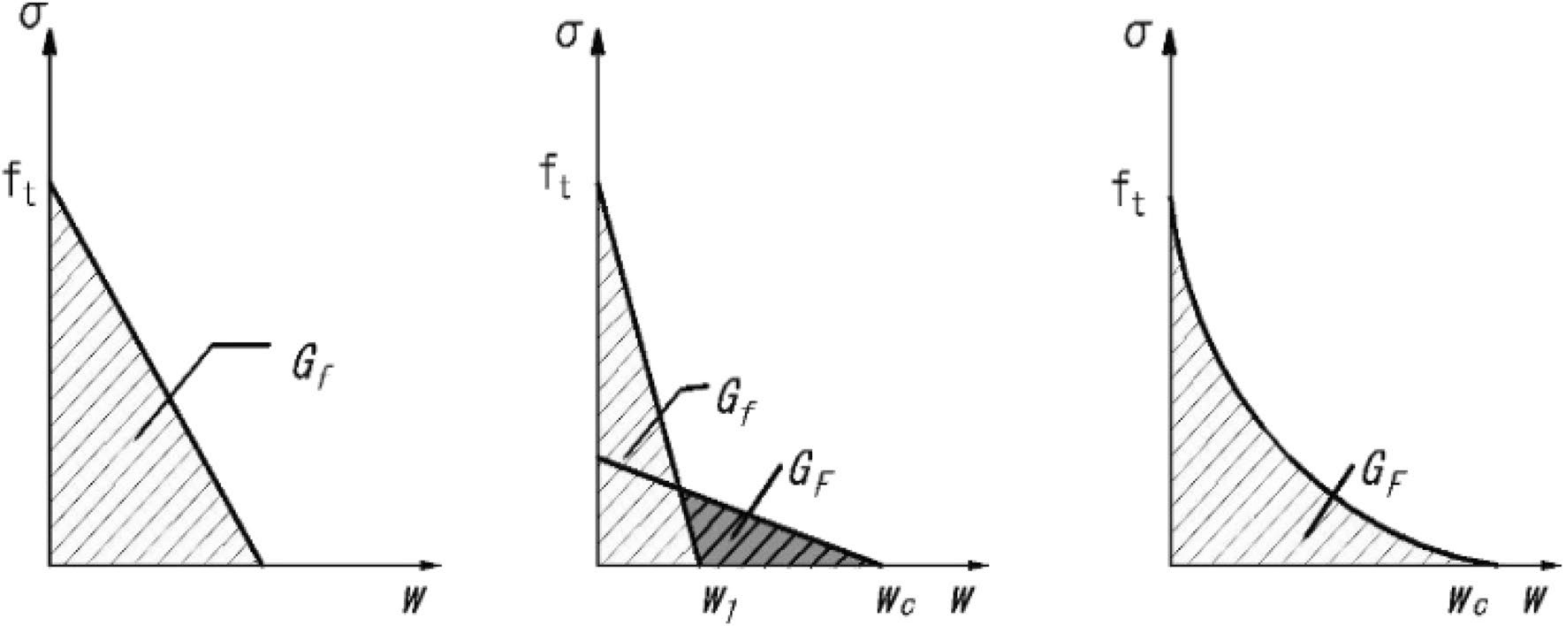
linear, bi-linear and exponential relations for tension stiffening.

**Figure 8:**
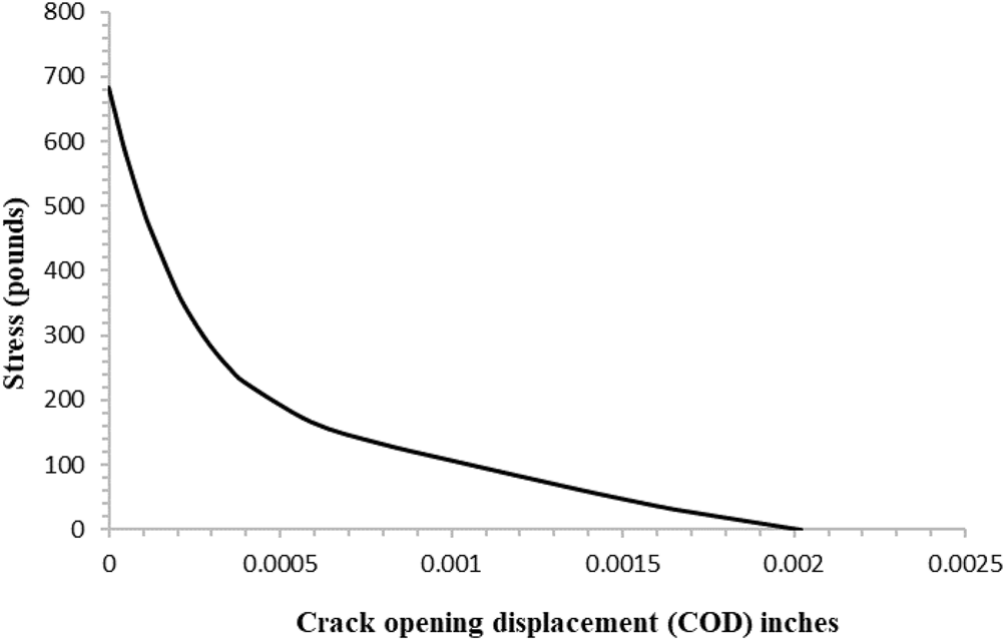
Crack-opening displacement vs Stress data used in the numerical model.

Concrete's tensile behavior is characterized by an initial elastic part that extends to its peak tensile strength, followed by a branch that softens. It is possible to simulate the softening branch of the tensile behavior using stress and cracking strain or a fracture energy-based model that employs the brittle fracture concept proposed by^28^. In the later model, a crack opening displacement and stress relation is used to simulate the concrete's tensile behavior. The crack opening displacement and stress relation can be represented with a linear, bilinear, or exponential relation using the fracture energy criterion, as illustrated in Fig. 7. Due to localized cracks that are encountered in cantilever structures, the crack opening displacement vs stress relation is used to model the post-peak tensile behavior of concrete. Stress–strain relations are found to be mesh-sensitive in case of localized cracking^22^ and therefore are avoided in modeling the post-peak tensile behavior of the concrete. The exponential relation given in^29^ is employed for the tension stiffening behavior. The equations (Eqs. (8) and (9)) for the post-peak tensile behavior are given below and shown graphically in Fig. 8.

8 $$\sigma =ft\left(\left(1+{\left(c1\frac{w}{wc}\right)}^{3}\right)exp\left(c2\frac{w}{wc}\right)-\frac{w}{wc}\left(1+{c1}^{3}\right)\mathrm{exp}(-c2)\right)$$

9 $$ {\text{c}}_{1} \, = \,3\,\,\,{\text{c}}_{2} \, = \,6.93.\,\,\,w_{c}  = 5.14\frac{{Gf}}{{ft}}. $$

### Quasi static analysis in Abaqus

Material degradation and failure can cause severe convergence difficulties in implicit analysis^22^. To avoid this, viscoplastic regularization is employed in the CDP; however, convergence analysis needs to be performed for the viscosity parameter in the Abaqus CDP. To avoid this, Abaqus explicit solver in which the convergence problem due to material degradation are avoided, is utilized. The explicit solver divides the loading application step in to finite number of small steps based on the stable time increment. It is therefore computationally inefficient to perform the test in its natural time scale in the Abaqus explicit. In order to perform the numerical test in accelerated manner Abaqus/explicit imposes a limit on the kinetic energy content of the model. For a test to be quasi-static, the inertial effects should be as minimum as possible. Therefore, the external work should be balanced by the internal energy of the system with minimal or no energy input from the inertial effects. Hence, for a test to be termed as quasi-static using the Abaqus/explicit solver the kinetic energy should not exceed 5–10% of the internal energy of the system throughout the whole process^22^.

### Parameters selection for the model

In order to carry out the analysis, multiple parameters needed to be investigated for the initial model. These include the mesh size, step time for the lateral load, and CDP parameters mainly dilation angle. A comprehensive table about the CDP parameters for different concrete structure are given in the^25^. As per^25^ the values mainly used for the eccentricity is 0.1, Kc is 0.667 and $\frac{{\sigma }_{b}}{{\sigma }_{c}}$ =1.16, which were also adopted for this study as well. For concrete structures a preferable value for the dialation angle is 35–40^25,30^, hence, initially a value of 40 was adopted for the model. A mesh size of 2 in. was initially selected to depict the major damage region properly since the damages observed experimentally were up to 6-in. height. A higher mesh size could overestimate the damage region. A step time of 60 was selected for the lateral loading step.

### Validation procedure

For the numerical validation two parameters were considered: load response curve and damages pattern. The load response curve of the model was compared with the experimental backbone curve as material properties and geometry similar to the experimental one and given in Table 1 were used for the model. Similarly, the damages pattern of the model was also compared with the experimentally observed damages.

## Results and discussions

### Validation of the numerical model

**Figure 9:**
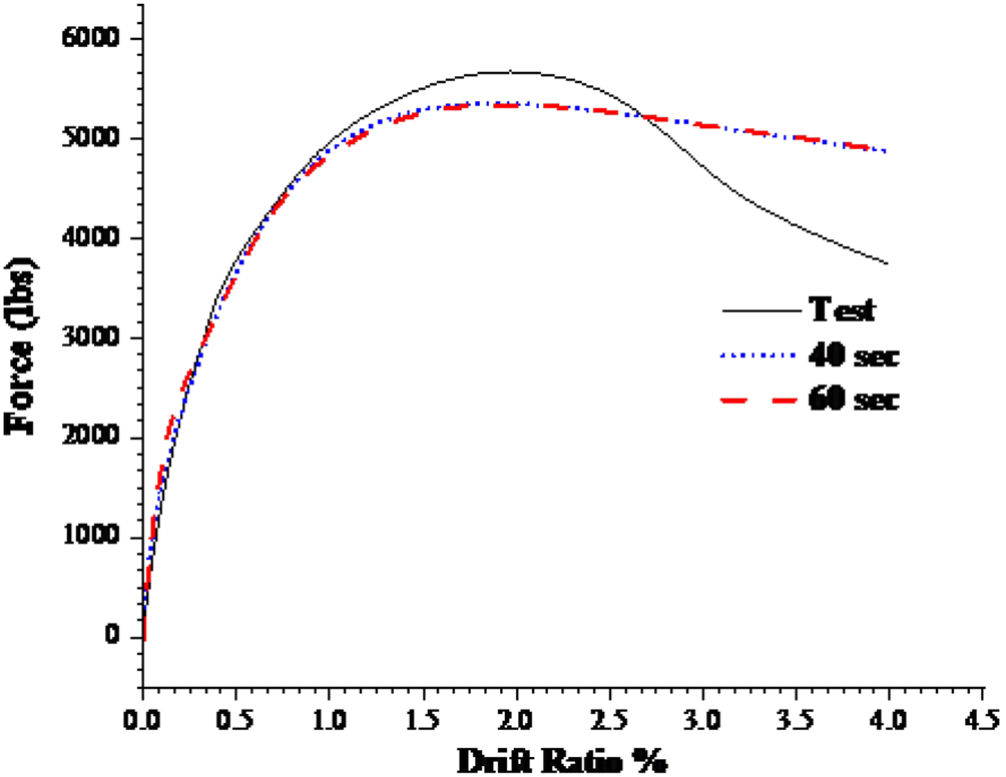
Load- deformation curves.

**Figure 10:**
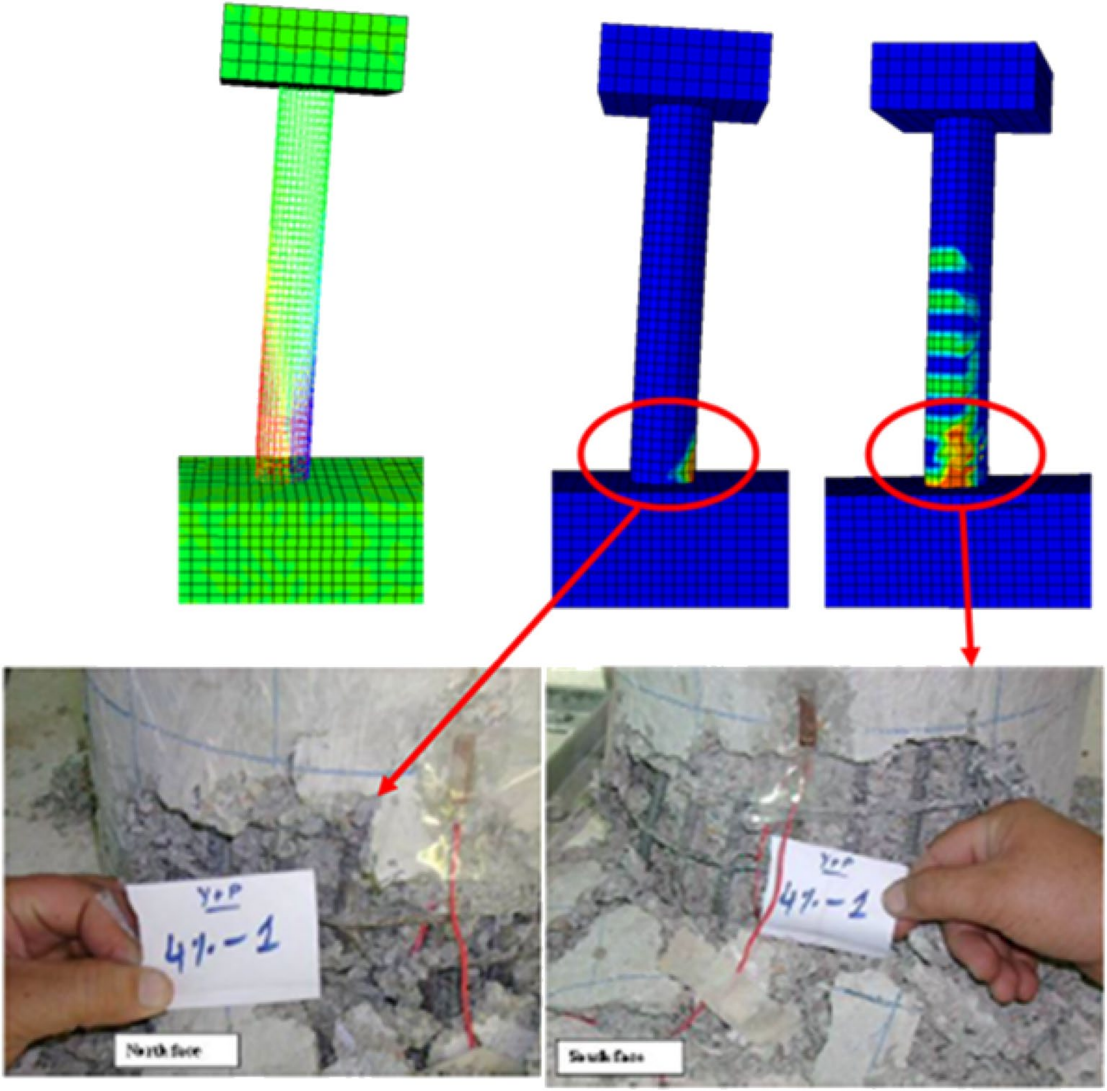
Damage comparison of the numerical and experimental column at 4% drift.

**Figure 11:**
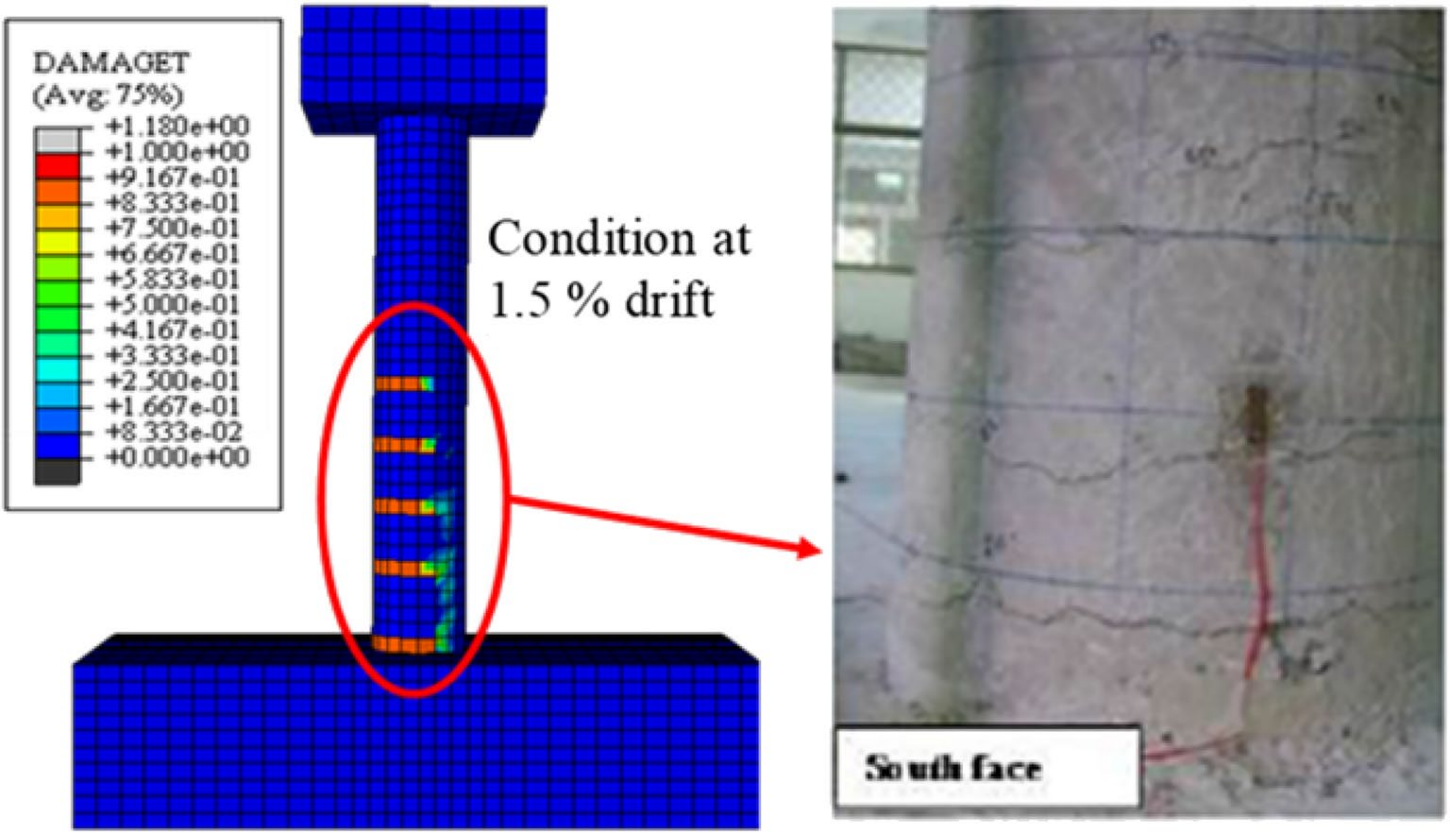
Damages at 1.5% drift compared with experimental damages.

The response of the initial model was compared with the experimental backbone curve and are given in the Fig. 9 and the damages are compared in the Figs. 10 and 11. The force–response curve of models follows an initial path which is close enough to the experimentally obtained force-response. The initial response of the systems is similar to the experimental behavior until 1% drift. This deviation may be attributed to the strain hardening effect in the rebar which were not taken in to account in the numerical model due to the application of the bi-linear elastoplastic curve. The first yield was observed at exactly 1% drift experimentally and approximately in the numerical scheme. The rebars in numerical model ceases to take any further load once yielded, however, in the real scenario the rebars can take load after yielding due to the strain hardening effect. In addition to this the difference between the strength and stiffness of the actual and analytical model, the actual being greater, also contribute towards this variation. After this point, the load response starts to deviate from the actual response. It is worth mentioning here that the initial part of the force response curve corresponds to the tensile damage. The effect of mesh on the initial portion is minimal due to fracture energy-based criterion being employed for the tension stiffening of concrete as can be seen in Fig. 9. The peak load observed during the experimental test (5750 pound) is greater than the numerically observed peak load (5340 pounds), which is almost 8% difference. This discrepancy may be attributed to the effect of strain hardening in rebars as well as the 12.5% difference between the analytical (2400 psi) and actual concrete strength (2680 psi) is also a contributing factor in this regard. The numerical model's maximum load corresponds with full rebar yielding on the tensile side, confirming that the model can no longer support additional loads after rebars have yielded. The failure in the compression face of the column is aligned with the post-peak behavior. This tendency is attributable to the slope of the concrete's falling branch in compression, which will be described later. Figures 10 and 11 display the damages determined during the numerical and experimental analysis. Figures 10 and 11 shows that the model's representation of damages is in good conformity with the experimental observation. Spalling phenomenon of concrete cannot be modeled due the continuum nature of the model. However, form the contour plots of the damages observed, it is evident that considerable damages are experienced by the model up to 6-in. height which is also the case in the experimental observation. The deviation in the post-peak region of the numerical backbone from the experimental one can be attributed to the softening branch of the compressive behavior of concrete and will be discussed in the subsequent section.

**Figure 12:**
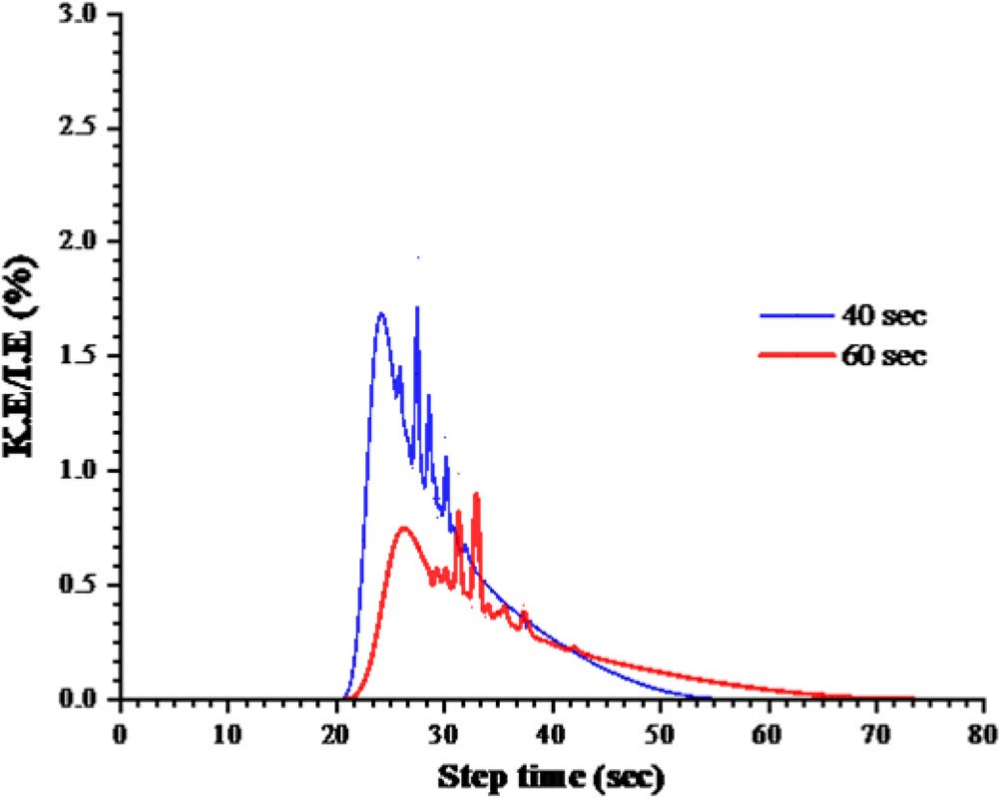
Energy content comparison for 40 s and 60 s step time.

### Effect of analysis step time

The loads on the numerical model were applied in the Abaqus using a two-step approach. In step-1 the superstructure loads were applied using the pressure type of loading with smooth amplitude having a step time of 20 s. Abaqus explicit solver utilizes the displacements, velocity and acceleration of the previous step to solve the equation of the current step. Hence, to minimize the inertial effect of the superimposed load application in step-1, 20 s step time with a smooth amplitude was selected which was observed to produce negligible inertial effect. The lateral load was applied on a reference point which corresponded to the load application point in the experimental test. For numerical efficiency further analysis was performed to select the minimum possible step for the lateral load application while taking the quasi-static nature of the test in consideration. Two step-times were used for the application of lateral load: 40 s and 60 s. Energy content of both these step-time is compared and are shown in Fig. 12. It is evident from the figure that the acceleration effects are more predominant in the 40 s time steps. The ratio of Kinetic energy to Internal energy is more than twice in 40 s step time. Furthermore, as the model parameters established initially were to be used for the further parametric analysis and given the uncertainty of the process, 60 s step time was deemed suitable for further investigations in this study. In contrast to the energy content the load response behavior is not affected by the given time steps as given in the Fig. 9.

**Figure 13:**
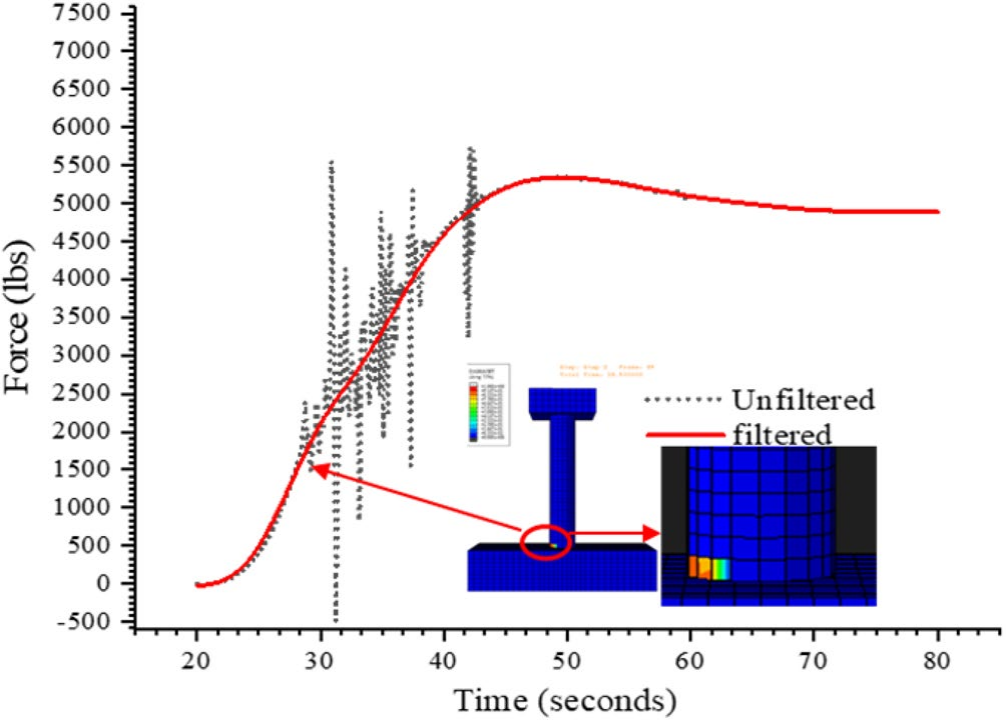
Filtration of the force-response curve.

Abaqus/explicit solver was initially developed for the dynamic analysis of the structures to avoid convergence difficulties that arises due to material failure in implicit analysis. Moreover, material failure as in case of tensile and compressive failure of concrete imparts dynamic oscillations to the system. In addition to this, the output sampling was carried out a frequency of 0.1 Hz. The difference in the output frequency and stable time increment (9.37508e−05 s) also contributes to the oscillations. The Butterworth filter in Abaqus is employed in the post processing phase of the modeling to remove the unwanted oscillations. The cutoff frequency of 0.1 Hz was selected due to similar output sampling frequency. Figure 13 shows the filtered force-response curve with a cutoff frequency of (0.1 Hz) using Butterworth filter. It is evident form the figure that 0.1 Hz cutoff frequency effectively removes the oscillations and gives a convincing overall behavior.

### Mesh sensitivity analysis

Meshing can significantly affect the computational time and results of a numerical study. For computational efficiency, it is necessary to conduct sensitivity analysis so that an optimum size and configuration of mesh is selected without compromising the numerical results. Load response behavior and damage patterns were the parameters used for the validation of optimum mesh size and configurations selection.

**Figure 14:**
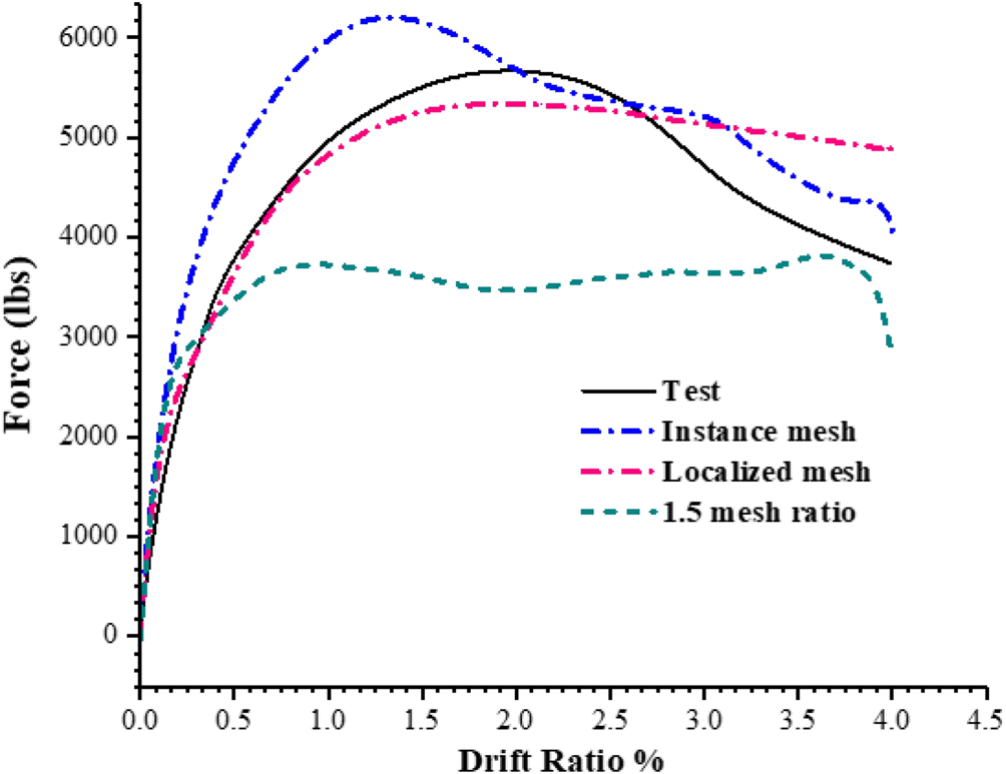
Load–response curve for different mesh configuration.

The interaction between the various surfaces is influenced by the mesh^31,32^. Therefore, the model was initially tested with a localized mesh configuration in which the column and the footing nearby the columns were discretized with the same mesh size which was used for the initial validation. To further investigate the effect of mesh two more configurations were used: a complete instance mesh, where the entire model was meshed at once and a mesh ratio of 1.5 between the footing and column mesh. A mesh size of 2-in. was used for all these configurations. The load response curves of all these mesh configurations are shown in Fig. 14.

The load response behavior of the instance mesh shows a significant deviation from the actual load response behavior. The peak load observed in the instance mesh configuration is close to 6207 lbs. which is more (8% greater) the actual test. The 1.5 mesh ratio configuration does not depict any convincing behavior. The peak load observed is 3728 lb. which is 35% less than the actual load. The trend of the load-response in the case of 1.5 mesh ratio almost follows a flat plateau after peak load and does not gives any convincing result. In contrast to these, the localized mesh shows a convincing behavior and follows the experimental curve very closely and the peak load is 5340 lbs. (8% difference). The peak load difference is same for the instance mesh and localized mesh, however the later shows a more overall close behavior.

**Figure 15:**
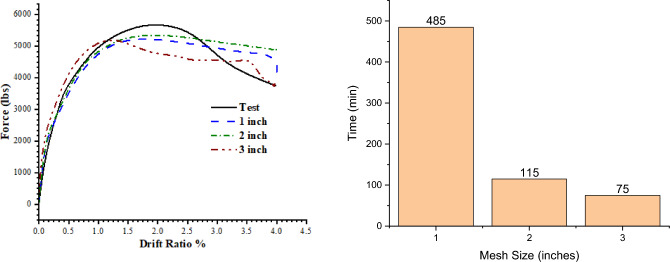
Load response curve for the multiple mesh size (left), computational time comparison for the multiple mesh size (right).

**Figure 16:**
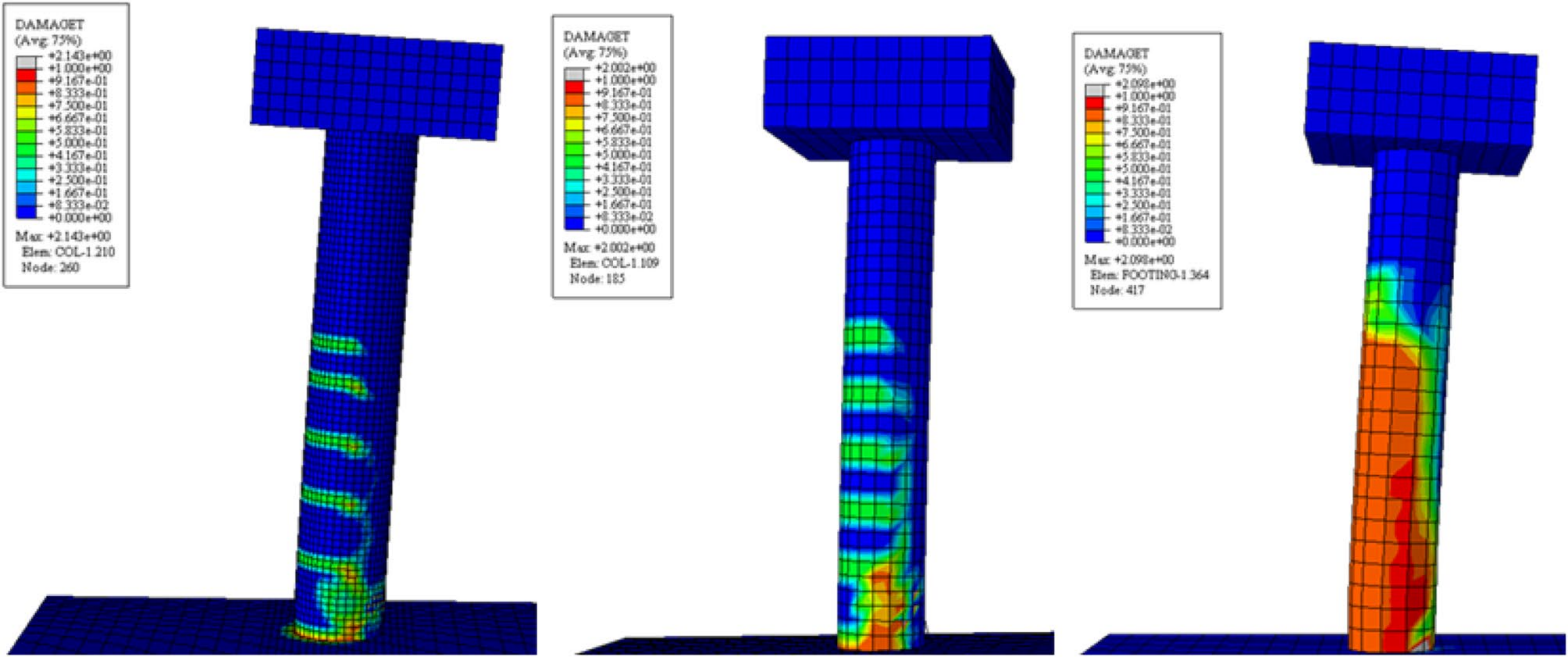
Damages observed during numerical model (from left, 1 in., 2 in. and 3 in.).

In addition to this, mesh size effects were also investigated. 1-in. 2-in. and 3-in. mesh sizes were used using the localized configuration scheme. Comparisons of computational time and load response behavior of the mesh size effects are given in the Fig. 15 and the tensile damages are given the Fig. 16. In contrast to the configuration, the mesh size gives similar peak loads. All the graphs follow the actual curve closely, however, the 3-in. mesh shows a steeper falling branch compared to the others. The damage pattern in case of the 3-in. mesh is overestimated, while the 1-in. mesh shows an underestimated damage region. The 2-in. mesh size gives results in good agreement with minimum computational effort, and it is used for further numerical analysis.

### Effect of stress–strain law of the behavior of concrete column

**Figure 17:**
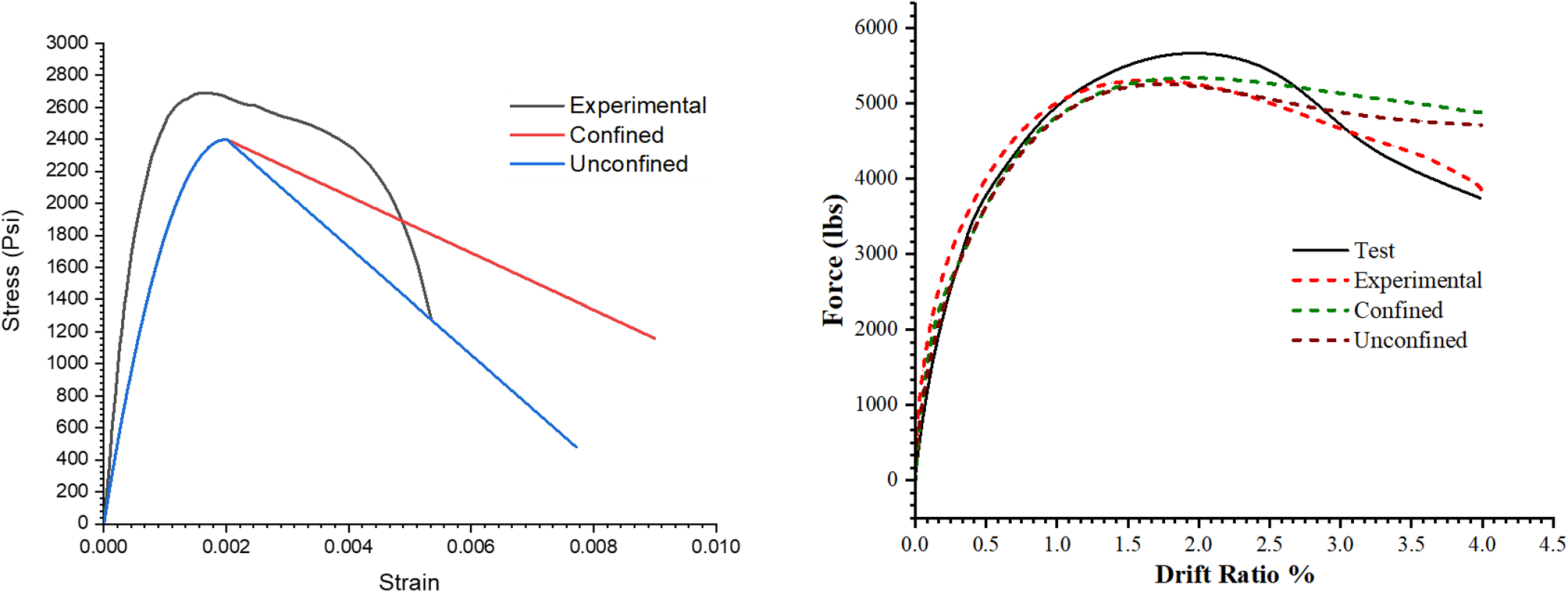
Stress–strain relation used (left). Load response of each of these stress–strain relation (right).

Abaqus CDP uses two hardening variables, these are the ${\widetilde{\varepsilon }}_{c}^{pl}$ and ${\widetilde{\varepsilon }}_{t}^{pl}$ compressive and tensile equivalent plastic strains . These variables are calculated by Abaqus from the stress–strain data input given to model the concrete behavior, thus, it is clear that the behavior of the numerical model is significantly affected by the concrete stress–strain law. Three stress–strain relations for concrete's compressive behavior were utilized to investigate this effect: experimental stress–strain relation and the confined and unconfined Kent and Park models illustrated in Fig. 17. The results of these analysis are also shown in Fig. 17. It is obvious from the load-response curve of given in Fig. 17 that the post peak behavior which is attributed to the failure on the compressive side is significantly affected by the softening branch of the stress-stain curve. The post-peak behavior obtained from the model with experimental stress–strain curve follows path very closed to the experimental backbone curve. Furthermore, the unconfined law has no significant effect on the force-response curve. This behavior is due to the reason that the in the experimental test 5-1mm diameter plain bars were used which have no significant confining properties.

**Figure 18:**
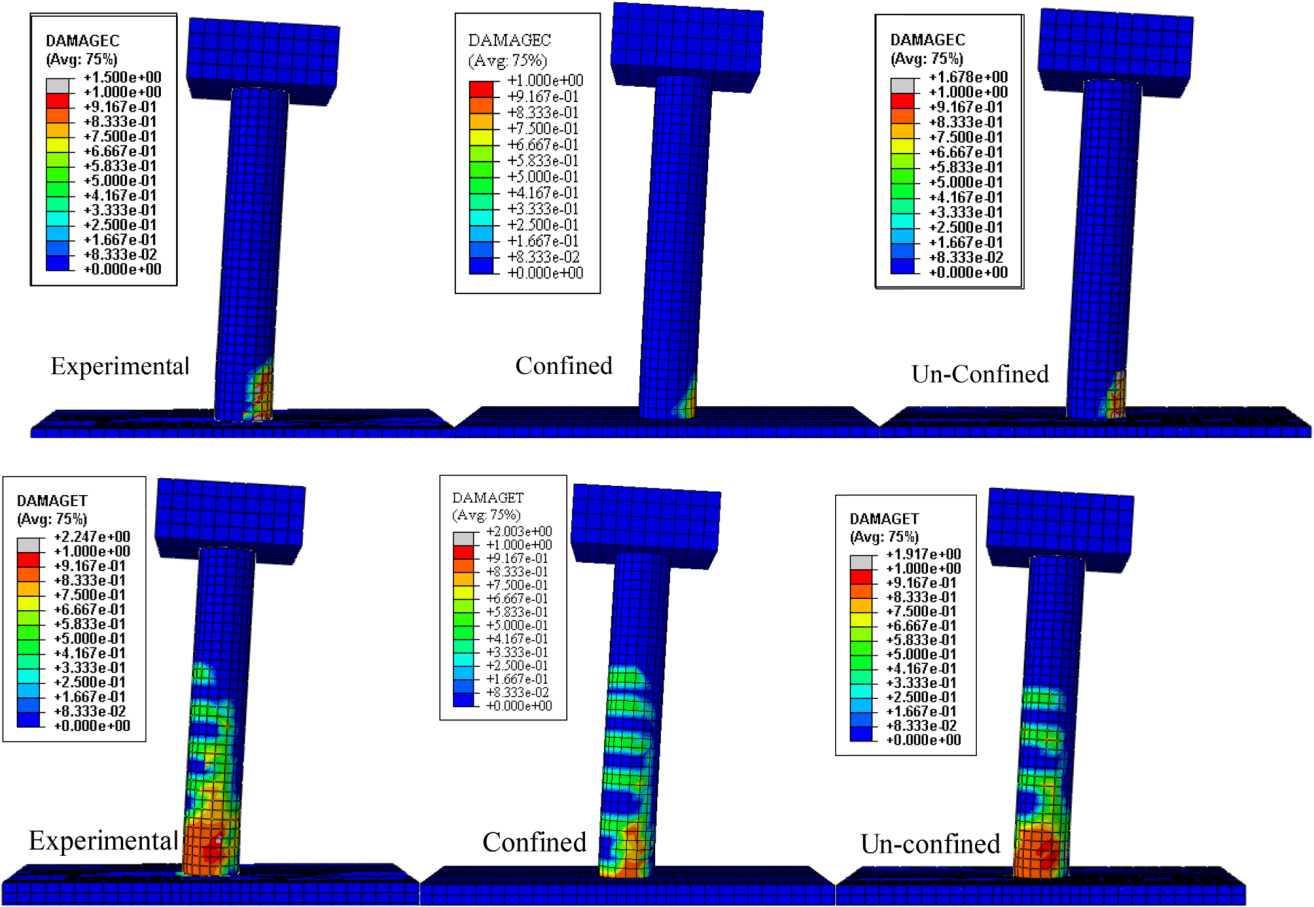
Compressive damage (above) and Tensile damage (below) for the three stress–strain relations.

It can be seen from the experimental backbone curve that there is a significant loss of load carrying capacity after the peak load. The same behavior is also observed in the numerical model with the experimental stress-strain relation. Damage comparison of all the three constitutive relation are also given in Fig. 18. The damage profiles of the actual concrete compressive stress–strain relation are close to the experimentally observed. The whole region within the 6 in. from the base of the column has experienced damages. Similarly, the unconfined relation has also more damaged portion than the confined one.

### Performance evaluation of the bridge column

**Figure 19:**
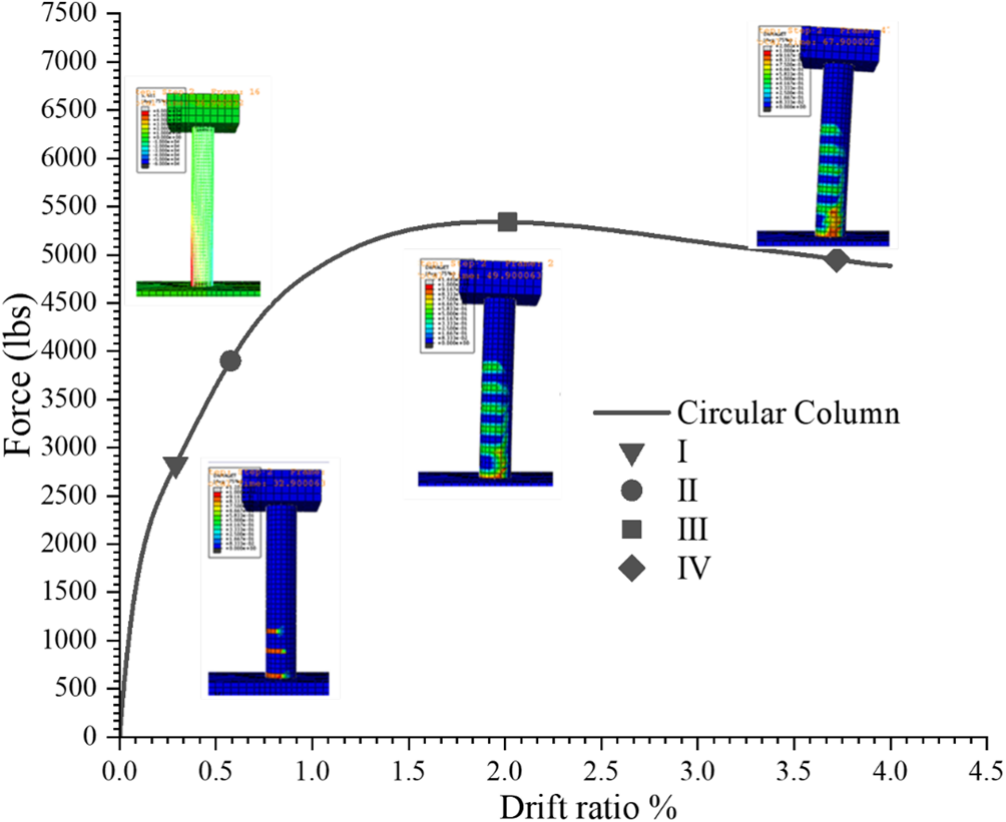
Performance level comparison of the single column bridge pier.

Hose and Seible^13^ developed a five-level performance and damage assessment criteria for the evaluation of bridge piers after an earthquake. The damages criteria is based on their socio- economic impact. These damage levels are then connected with the performance level through qualitative descriptions of damages for detail see^13^. Same performance evaluation criteria^13^ have been used for the assessment as given in Table 3 for the performance comparison of the experimental and numerical pier. The performance level 5 given in the Table 3 cannot be evaluated due the inability of truss elements to model buckling. Figure 19 shows the performance comparisons of numerically analyzed column. When compared with the experimentally observed performance level qualitative description given in^7^, only performance level 3 comes in good agreement with the test column. Performance level 1, 2 and 4 have significant difference in terms of drift ratio as well as load. This difference in the performance levels is attributed to the difference in the concrete analytical and real behavior as well as the strain hardening effect of rebar. The initial stiffness of the experimental stress–strain relation as shown in Fig. 17 is higher than the analytical one which may be the cause of the performance level 1 and 2 to be observed with a higher load value. Similarly, the difference in level 4, which is observed at a higher load value numerically, is due to the softening branch of analytical stress–strain curve which has a comparatively mild slope than the experimental stress–strain.

**Table 3:** Performance evaluation criteria^13^.

Level	Level of performance	Qualitative description of performance
I	Cracking	Initiation of hairline cracks
II	Yielding	Theoretical initial yield of main reinforcement
III	Initiation OF LOCAL MECHANISM	onset of inelastic deformation. Beginning of concrete spalling Diagonal cracks development
IV	Complete development of local mechanism	Widening of crack widths /spalling of concrete over full local mechanism region
V	Degradation of strength	Buckling of longitudinal reinforcement. Rupture of transverse reinforcement. Core concrete crushing

### Effect of dilation angle

**Figure 20:**
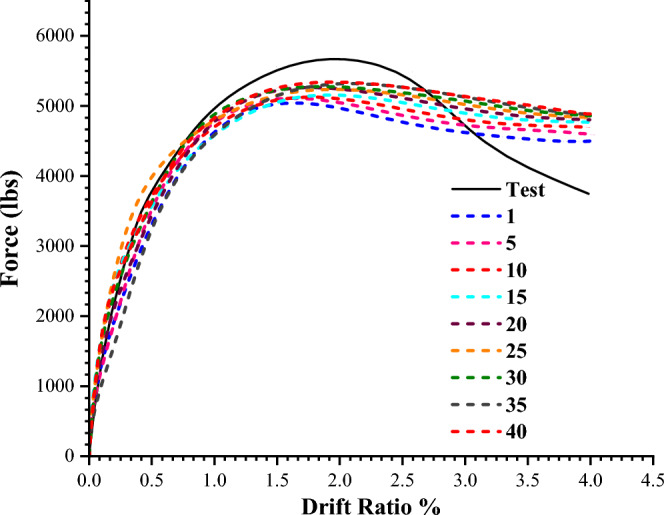
Load-response comparison for the different dilation angles.

The change in the material's volume as a result of failure is referred to as dilation. It is one of the variables that contribute to the plastic potential function of the CDP. Regarding the values of the dilation angle, there is no agreement in the literature; different researchers have chosen different values for different structures. For the analysis of a reinforced concrete frame, Alfarah et al.^33^ employed a value of 13. Tong et al.^19^ Employed 20 to represent the cyclic behavior of a concrete column. The non-linear behavior of a three-dimensional beam was studied by^34^ using dilation angles ranging from 20 to 50. Wosatko et al.^26^ studied the impacts of dilatancy on punching shear demand using dilation angles ranging from 5 to 55. In order to investigate the effect of the dilation angle on the behavior of the model, a dilation angle of 40 was first employed in this study. Subsequent analysis was then done on angles ranging from 1 to 40. The analysis' findings are presented in Fig. 20. Figure 20 clearly shows that the relation between load and deformation is unaffected by the dilation angle. Furthermore, the experimental curve is more closely followed by the 40 dilation angle than by the other angles. Therefore, the effects of other parameters were investigated using the dilation angle of 40.

### Effect of concrete strength

Employing the computational method used previously in the sections above, bridge pier with concrete strengths of 1800 and 4000 psi were simulated to determine the effect of concrete strength on the performance. A survey by^4^ revealed that existing bridge piers had concrete with a strength as low as 1800 psi, hence, 1800 psi strength concrete was selected as one of the target concrete strength for the parametric analysis. The study also investigates 4000 psi strength concrete, which is frequently utilized in reinforced concrete piers. The ACI (318, 2002) equation was used to estimate the concrete's modulus of elasticity. Similar CDP parameters to those of the 2400 psi concrete were utilized to simulate concrete behavior under compression using the confined Kent and Park model.

**Figure 21:**
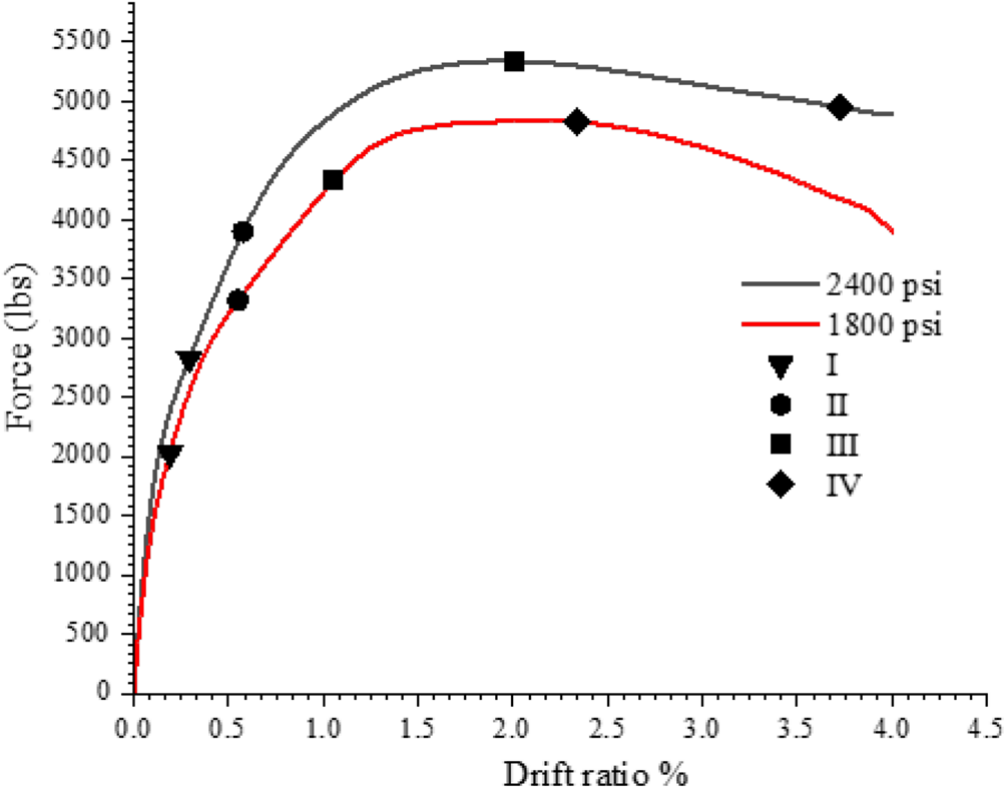
Performance level comparison for the 1800 and 2400 psi concrete.

**Figure 22:**
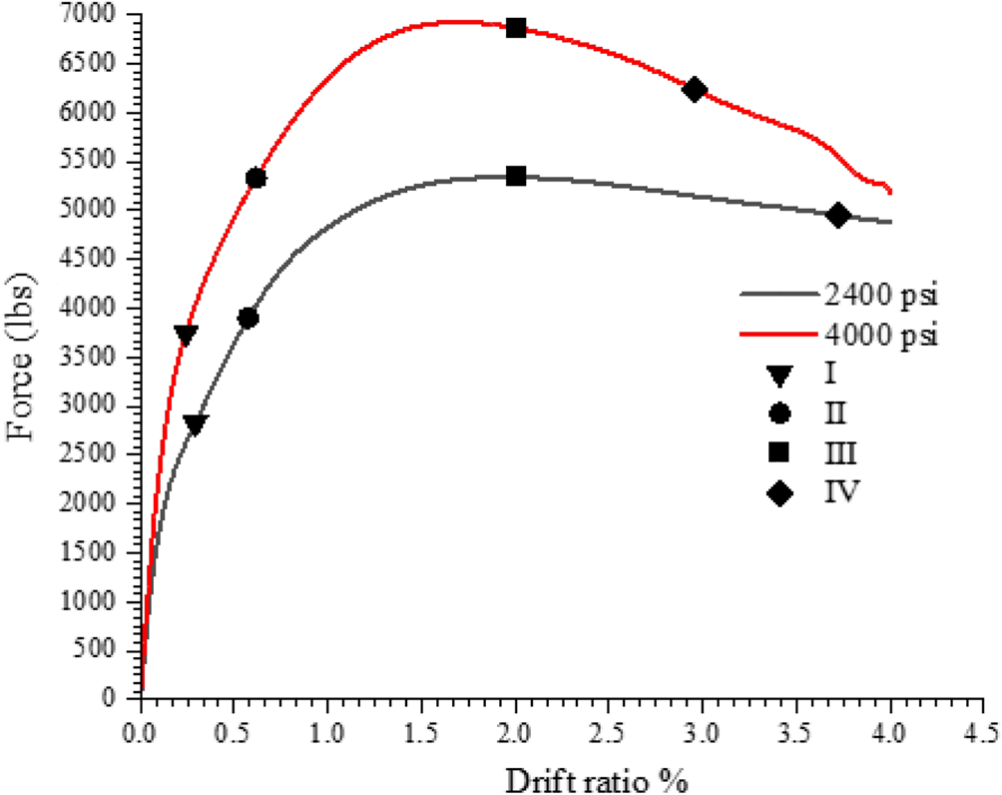
Performance level comparison for the 4000 and 2400 psi concrete.

The columns' performance levels were compared to the numerically tested circular column with a concrete strength of 2400 psi, illustrated in Figs. 21 and 22. The data clearly shows that the concrete strength has a considerable impact on the performance levels of the column. The performance levels 1 and 2 occur at close drift levels, but there is a large variation between the 1800 and 2400 psi concrete performance levels 3 and 4. With 1800 psi concrete, a reduced peak load was obtained, confirming the fact that concrete strength significantly affects the column's performance and peak load. The performance levels were obtained at drift ratios that were equivalent to those of the 2400, though with higher loads in the case 4000 psi. The peak load of the 4000 psi concrete model is significantly higher. With 4000 psi concrete, performance level 4 was attained with less drift than with 2400 psi. In the instance of the 4000 psi concrete, the system's early reaction is also stiffer, which is also a characteristic of concrete; higher strength concrete has higher initial stiffness.

### Effect of geometry

**Figure 23:**
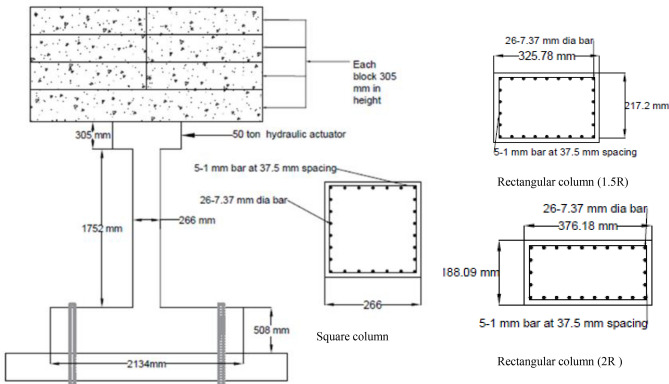
Geometries of the bridge piers.

The influence on level of performance due to the variation in geometric shape of the column was explored using the given numerical scheme. Based on their equal cross sectional areas, the geometries of a single square and two rectangular columns were chosen at random. Initially, a 266 mm square column was modeled. A length to breadth ratio of 1.5 and 2 was used in the modeling of the rectangular columns. We will also use the terms 1.5R and 2R to refer to the rectangular columns having length to width ratio of 1.5 and 2, respectively. A 2400 psi concrete strength was chosen, and the initial model's longitudinal and transverse reinforcement was maintained. Instead of spirals, rectangular stirrups with spacing corresponding to the spiral's pitch in the initial circular column were modeled. Figure 23 provides the geometry and reinforcement information. To maximize efficiency of model, the pedestal and column base were modeled utilizing simple elastic concrete behavior. Since these components responded elastically both during the experimental and numerical tests.

**Figure 24:**
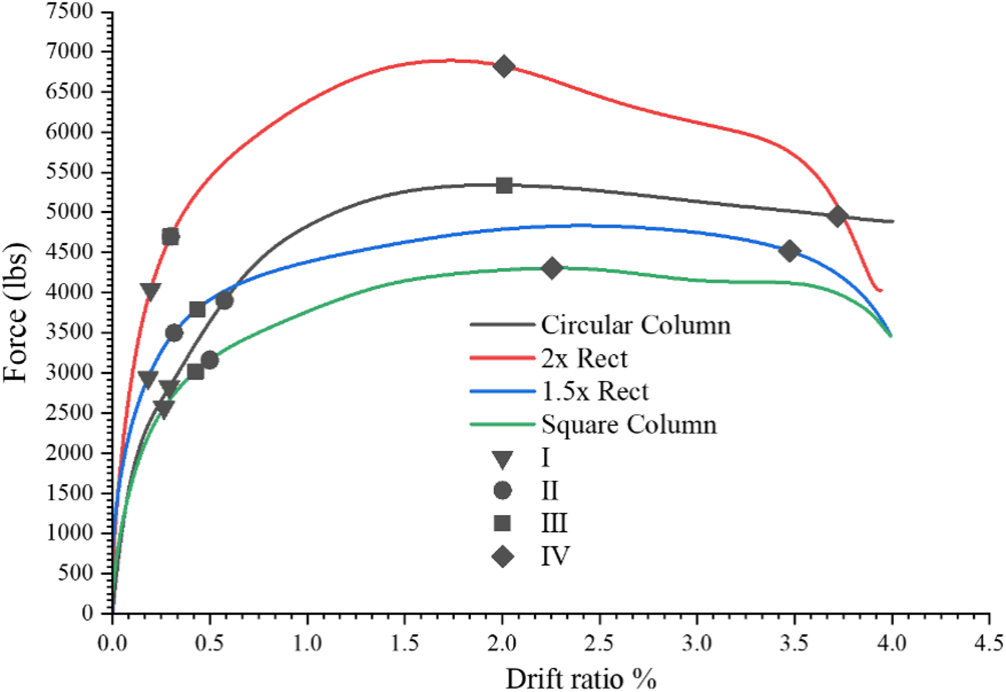
Performance level comparison of various column geometries and circular column.

Figure 24 demonstrates the square's and the circular column's performance level. In comparison to the circular column, the first three performance levels in the square column are attained with minimal drift. Performance Level 3—which is associated with concrete diagonal cracks—was attained first, then Performance Level 2 (yielding of rebars). In contrast to the circular column's 3.5% drift, the performance level 4 was obtained with only 2% drift. Additionally, the damage pattern that was identified during the computer modeling is not totally consistent with the typical behavior of columns. The 1.5R column exhibited a similar pattern to that displayed by the square column as shown in Fig. 24. Hence, at lower drift levels, the first three performance levels were attained. At a 2.5 drift ratio, the performance level 4 was reached. In contrast to the circular column, the column 2R achieves performance level 1 at a larger load and about the same drift level shown in Fig. 24. Rebar yielding and concrete spalling happened simultaneously since performance levels 2 and 3 in column 2R were observed to occur at the same drift level and load. Performance level 4 was attained with a greater load and an almost 2% drift. It is evident that the column's initial response is influenced by the stiffness of the column in the loading direction. In the initial part of the load response, 1.5R and 2R columns had stiffer responses compared to the circular column. Additionally, the column 2R demonstrated a larger peak load as a result of increased stiffness (twice of the circular). The damage pattern observed in square and rectangular shapes differs from what is generally observed. This odd behavior is the result of the fact that the dimensions of the columns were chosen at random without any actual design or undertaking any prior analysis for the selection of CDP model variables for square and rectangular geometries.

**Figure 25:**
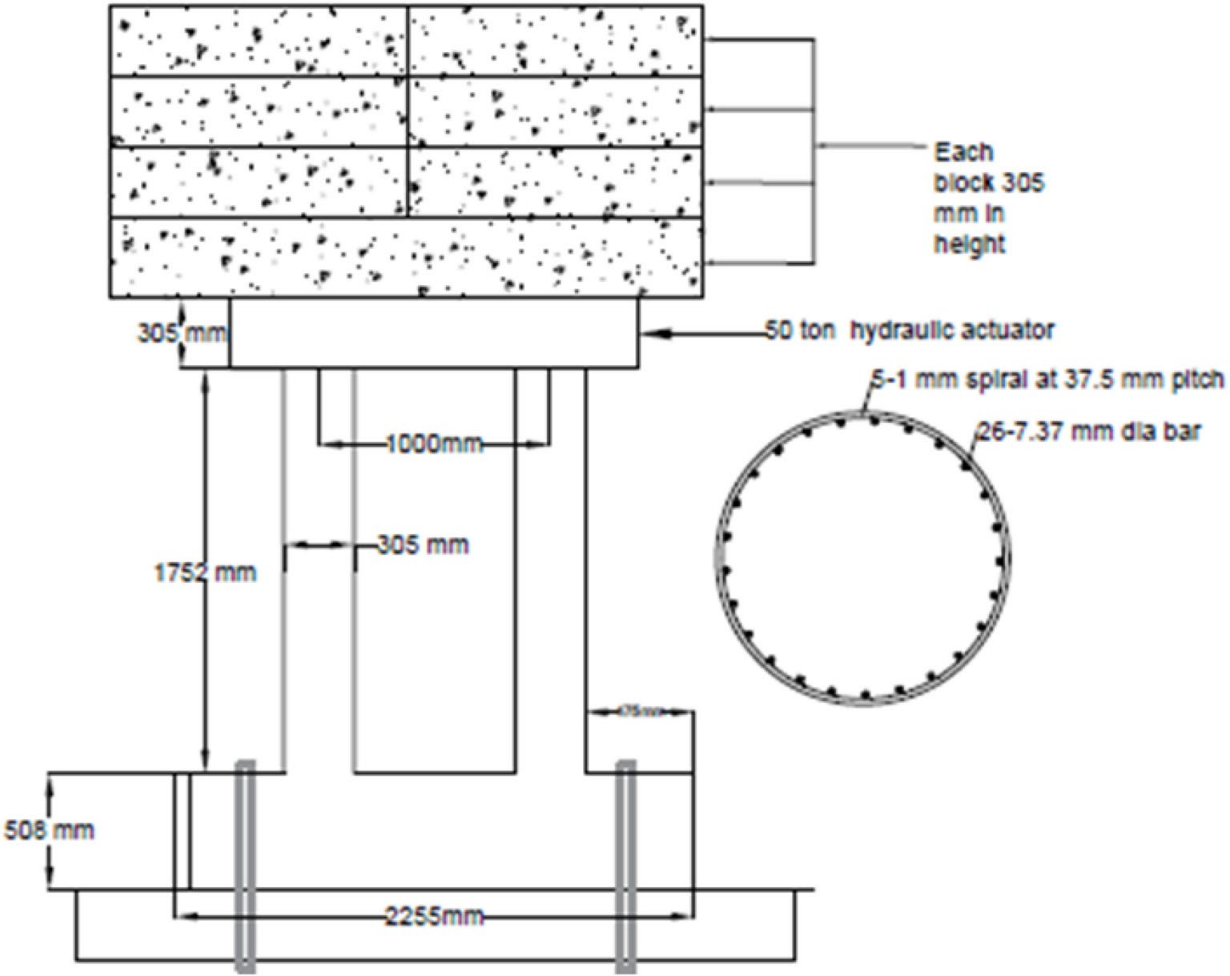
Geometry of twin column.

**Figure 26:**
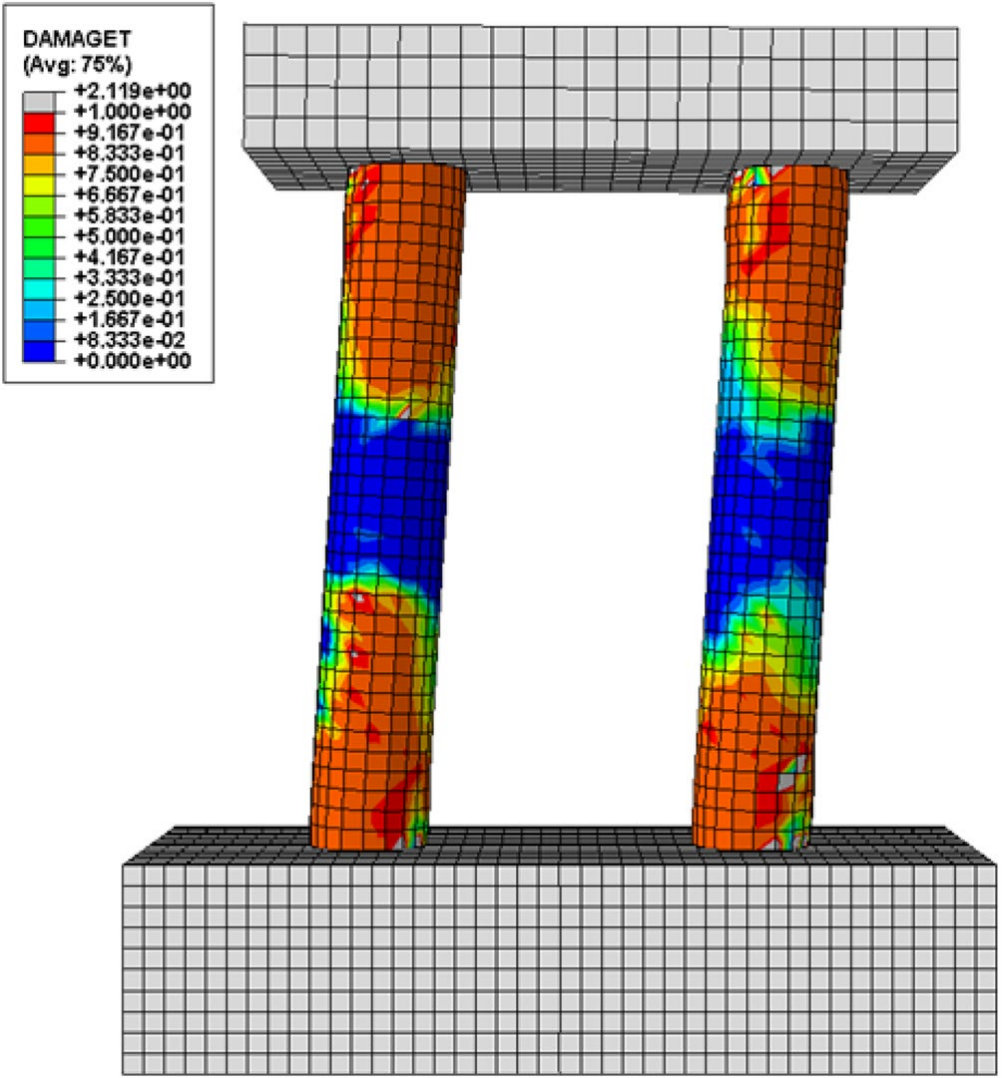
Tensile damages observed in twin column.

**Figure 27:**
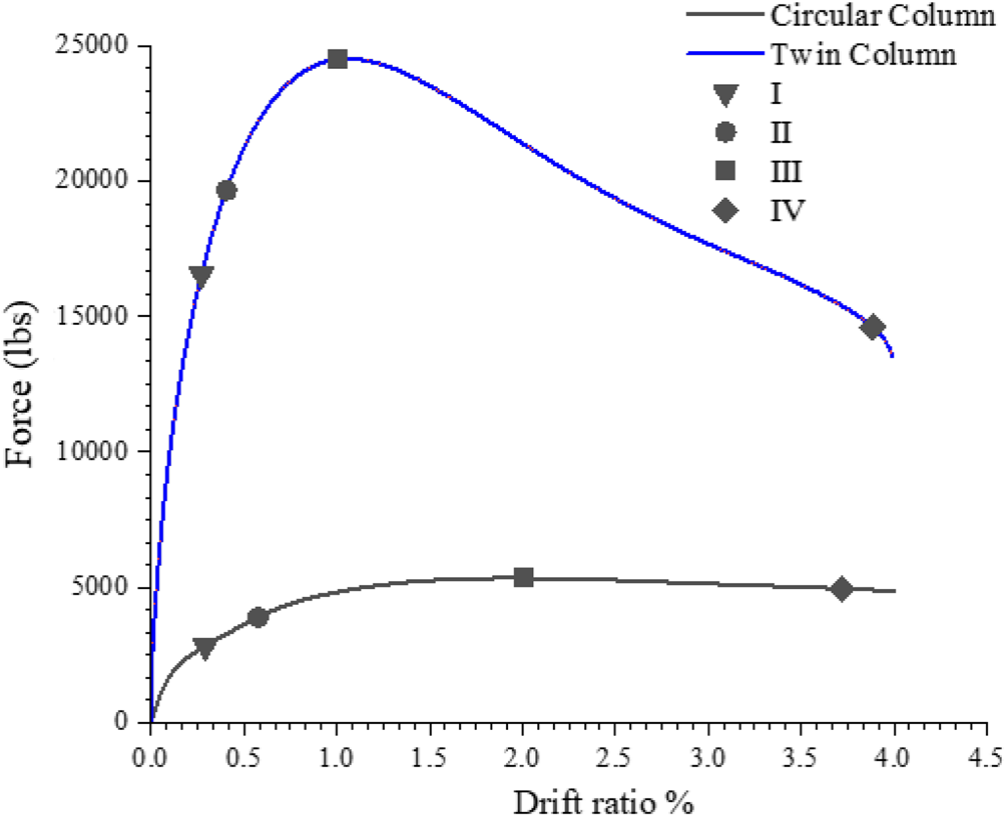
Performance level comparison for circular and twin column.

To investigate the behavior of multiple column bridge piers under lateral loads, twin column configuration in addition to the single column configuration is also modeled. The twin column configuration uses the same column dimensions and reinforcement details as the single circular column as seen in Fig. 25. The space between the columns is determined based on a directory of bridge designs that is locally available. The loads placed on the superstructure are double that of a single column. Loads were applied to the column solely in the transverse direction. Figure 27 depicts the twin column configuration's load response curve; Fig. 26 depicts the damages. The damage pattern depicted in Fig. 26 is compatible with the behavior of multiple column frames that is commonly noticed, i.e. the formation of several plastic hinges. As illustrated in Fig. 27, the performance levels for multiple column configuration are achieved at around the same drift level but with significantly higher loads. In comparison to a single column, the peak load is five times higher. The multiple plastic hinge (4) formations is what really induce the higher peak loads. Kim et al.^35^ investigation also confirms the formation of four plastic hinges in the lateral directions, even though the investigation of^35^ was based on circular columns with different material properties and dimensions.

### Conclusions and recommendations

In this paper, a computational method was suggested utilizing the Abaqus explicit solver, for the nonlinear behavior of bridge piers. The main parameters evaluated in this study are the load-response behavior and damages in RC bridge piers. The created model comprises a single circular column that is 146 in. long and 12 in. in diameter. In the model, concrete with a 2400 psi strength was employed. Using force-response relation, the model was calibrated against an experimental test. Then, parametric analyses were performed on the calibrated model in order to determine the impact of the stress–strain law, compressive strength, geometry, and configuration on the behavior of bridge pier. Following conclusions were made in this study.

- In contrast to macro scale models, the scheme suggested in this study allows for a complete non-linear analysis and explicitly provides us with the peak load, rebar yielding, and pattern of damage.
- The slope of the falling branch of the compression stress–strain curve controls post-peak behavior. Actual stress–strain data for concrete from an experimental test produces results that are comparably more consistent than those from an analytical stress–strain relation. As shown, the falling branch of the load response curve form the actual stress–strain relation is more close to the actual load-response than the rest of the concrete models. The difference between the final failure load is almost 25% between the actual test and models with analytical relation, while this difference is negligible in case of the model with the experimental stress–strain relation.
- Mesh size and configurations should be thoroughly investigated. As shown in this study, these factors have significant influence on the overall damages profile as well as load response behavior.
- It was shown that when utilizing an explicit approach, the dilation angle had no noticeable effect on the genearl behavior of model, however, an angle of 40 gives a curve that follows the actual one more closely.
- The suggested computational method does not produce a plausible overall behavior in terms of the damages or the overall performance for geometries other than circular forms and the parameters developed for the circular geometry should be modified based on sensitivity analysis for other geometries.
- Peak loads and performance levels are significantly influenced by the strength of the concrete. Peak loads for concrete with a 4000 psi strength increased by about 27% while those for concrete with an 1800 psi strength decreased by 15%.
- The twin column arrangement exhibits high levels of performance and peak load that is almost five times higher (25,000 lbs as compared to 5350 lbs of the single circular column) than that of a single column because of the production of multiple plastic hinges. However, due to similar geometric and material characteristics used for both the twin column and single circular column, higher ductility cannot be achieved.
- Concrete's behavior is substantially impacted by the stress–strain law; therefore, experimental stress–strain data should be employed, if available. It is possible to prevent difference in the load-deformation curve by using strain hardening relations for rebar. In order to assess performance level 5, the present explicit scheme used truss elements to imitate rebars; however, beam elements should be taken into account in place of truss elements to evaluate performance level 5. While using the current computational scheme, sufficient sensitivity analysis should be done for geometries other than circular shapes.

## Data Availability

The data used in the manuscript would be available upon reasonable request to the corresponding author.
